# Drivers of coral reef marine protected area performance

**DOI:** 10.1371/journal.pone.0179394

**Published:** 2017-06-23

**Authors:** Venetia Alexa Hargreaves-Allen, Susana Mourato, Eleanor Jane Milner-Gulland

**Affiliations:** 1 Centre for Environmental Policy, Imperial College, London, United Kingdom; 2 Department of Geography and Environment, London School of Economics and Political Science, London, United Kingdom; 3 Department of Life Sciences, Imperial College London, Silwood Park, Ascot, United Kingdom; 4 University of Oxford, Department of Zoology, Oxford, United Kingdom; Leibniz Center for Tropical Marine Ecology, GERMANY

## Abstract

Coral reefs are severely threatened and a principal strategy for their conservation is marine protected areas (MPAs). However the drivers of MPA performance are complex and there are likely to be trade-offs between different types of performance (e.g. conservation or welfare related outcomes). We compiled a global dataset from expert knowledge for 76 coral reef MPAs in 33 countries and identified a set of performance measures reflecting ecological and socio-economic outcomes, achievement of aims and reduction of threats, using spatial or temporal comparisons wherever possible. We wanted to test the extent to which distinct types of performance occurred simultaneously, understood as win-win outcomes. Although certain performance measures were correlated, most were not, suggesting trade-offs that limit the usefulness of composite performance scores. Hypotheses were generated as to the impact of MPA features, aims, location, management and contextual variables on MPA performance from the literature. A multivariate analysis was used to test hypotheses as to the relative importance of these “drivers” on eight uncorrelated performance measures. The analysis supported some hypotheses (e.g. benefit provision for the local community improved performance), but not others (e.g. higher overall budget and more research activity did not). Factors endogenous to the MPA (such as size of the no-take area) were generally more significant drivers of performance than exogenous ones (such as national GDP). Different types of performance were associated with different drivers, exposing the trade-offs inherent in management decisions. The study suggests that managers are able to influence MPA performance in spite of external threats and could inform adaptive management by providing an approach to test for the effects of MPA features and management actions in different contexts and so to inform decisions for allocation of effort or funds to achieve specific goals.

## Introduction

Marine Protected Areas (MPAs) are one of the principal tools for marine conservation and continue to proliferate due to ambitious international targets for the protection of marine ecosystems, [1–3]. The primary aim of most MPAs is to improve the ecological condition of an area in relation to its fisheries, habitats or biodiversity [4]. However, most MPAs also have socio-economic and governance objectives, such as increasing employment or stakeholder representation or decreasing conflict [5–7]. Conservation interventions act on social-ecological systems, in which interactions and feedbacks between social and ecological changes are inevitable [8, 9]. Yet the degree to which socio-economic and ecological outcomes can be achieved simultaneously is still contested, with some studies finding synergies between various distinct outcomes or “win-win” scenarios [10–13], some finding trade-offs necessary [1, 6, 14] and others still finding complex relationships [15–17].

Evaluations of MPA performance have documented large variations in ecological outcomes [18–20]. Many MPAs are failing to meet their aims and the majority of reefs, including those inside MPAs, remain threatened [1, 21–23]. Studies aiming to elucidate the drivers of this variation in performance have used a variety of approaches, for example an in depth qualitative analysis of 56 reef-related management projects to establish lessons learnt from success and failure [24], or correlations between community and management related variables and composite performance components [25]. Typically such analyses have focused on specific types of MPAs [26, 27] or those in a single country or region [25, 28, 29]. Global MPA studies have typically targeted ecological performance measures at many sites or multi-disciplinary data collected at fewer sites. A critical first global assessment of coral reef effectiveness determined the area of coral reefs within MPAs which were likely to provide effective protection as they fulfilled adequate criteria related to features and regulations [22]. Another global study assessed the influence of five MPA features (no-take area, enforcement, age, size and isolation) on eight fish community metrics calculated using field survey data at 87 MPAs [30]. A meta-analysis of coral cover surveys over several decades inside and outside 310 MPAs demonstrated what while coral cover inside MPAs remained constant, those outside had declined [20]. Another study conducted exploratory trend analyses on MPA effectiveness indicator data collected worldwide using a popular methodology, but the analysis was limited by the small sample size (n = 24) and lack of comparability of data generated [31]. A study in the wider Caribbean used social and ecological data from a rapid assessment of 31 MPAs and their associated human communities to investigates the extent to which MPAs are making progress toward their stated social and ecological objectives [17]. Most recently, a global analysis tested MPA performance compared to matched sites outside MPAs for a single metric; fish biomass [32]. The authors tested for relationships between certain management inputs and biomass increases at 62 MPAs and found that staff and budget capacity were the strongest predictors. Cross-site comparisons of performance have been complicated by numerous distinct methodologies with different goals and levels of robustness and comprehensiveness inconsistencies both in the definition of performance (also referred to as "success" or “effectiveness”) and in the indicators applied [33, 34], the use of composite scores, and limited availability of high quality data [9, 27, 35]. This has also limited the feasibility and usefulness of meta-analyses to elucidate performance [31, 36].

Evaluation of standardised, relevant, comprehensive and attainable management performance measures, including both socio-economic and ecological outcomes related to MPA goals, has been recognised as critical to adaptive management [31, 37, 38]. Such evaluations help us to better understand the conditions under which MPAs can achieve desired outcomes [39]. They can also inform budget allocations [16, 40], promote accountability and track progress towards broader conservation goals [34, 41]. A global analysis can supplement localised or ecologically focused studies and test widespread assumptions about the effect of specific inputs on performance, such as the importance of funding [32, 42] or education [24, 43]. It could also help to clarify the effect of certain variables on performance, including those that have been found to have different effects in different studies or in different MPAs, including MPA size and age [44], as well as those that have non-linear causal relationships with outcomes. For example, increased tourism can lead both to increased revenues, employment and compliance [45, 46] and also to environmental damage [47, 48], inequitable benefits [9, 49] and conflict [50, 51].

It is also instructive to explore the relative importance of ecological and socio-economic drivers that are endogenous, i.e. within the control of management, and global and contextual (exogenous) factors, e.g. national gross domestic product. Several studies have concluded that endogenous factors explain more variation in conservation outcomes than large-scale contextual factors [6, 11, 52]. Other research stresses the extensive influence of contextual factors on MPA outcomes [21, 53–55] or suggests that social factors determine performance at least as much as biophysical ones [51, 56]. The relative importance of contextual drivers on outcomes has critical implications for management, since management can only influence endogenous variables [57].

Hargreaves-Allen et al. [58] used a globally representative sample of self-report surveys from 76 coral reef MPAs in 33 countries, with 2005 as the reference year. Responses were used to identify a comprehensive but constrained set of performance measures, employing spatial (inside-outside) and temporal (before-after) comparisons where possible, to assess the extent to which coral reef MPAs fulfilled conservation and development goals and criteria. Here we use the same dataset, to explore the relationships between different types of ecological and socio-economic performance, and whether they are correlated or trade-offs exist between them. We then use multivariate approaches to examine which explanatory variables, including physical and governance features, management actions and local contexts, are associated with the different types of outcome and managers' overall perceptions of success. With these analyses, we tease apart the relative importance of endogenous MPA features and management actions and exogenous contextual factors in driving performance, considering both socio-economic and ecological drivers for distinct types of performance related measures. This is to the best of our knowledge the first time that a global dataset using a single effectiveness evaluation methodology has been used to test whether different types of coral reef MPA performance outcomes are correlated and employed a quasi-experimental approach using a multivariate analysis to test hypotheses as to the drivers of each distinct type of performance.

## Methods

### Survey and sampling approach

Site level data with adequate detail and breadth to evaluate MPA performance and provide information on potentially explanatory variables is not available at a global level, as the cost and effort would be prohibitive. We therefore designed questionnaire survey that relied on expert scoring (see S1 Text for full survey), which is regularly used in assessments at this scale [22, 59–61]. This cost effective approach generates comparable standardized qualitative and quantitative data by drawing on the perspective and field experience of experts with personal knowledge of each site, which can be repeated at low cost [62]. We incorporated performance related questions from several other popular methodologies and reviews [63–66]. We also gathered data on a wide range of variables that were expected to influence performance, all relating to 2005.

Here we use the definition of MPA as "coastal or oceanic management areas designed to conserve ecosystems together with their functions and resources” [67]. This included areas with diverse goals, features and management actions, which provided adequate heterogeneity to explore the effects of these variables on performance, by deliberately including potentially confounding factors [68]. We limited our population to MPAs containing an area of coral reef, as including all types of MPAs would have generated too much variability to undertake multiple regression analyses without very large sample sizes. The survey was extensively publicized in newsletters, MPA publications and a website and respondents were targeted at management symposiums, training workshops and through direct email correspondence. Respondents were self-selected and a snowballing approach was used with further contact information gleaned from NGOs, learning networks and other respondents.

There were approximately 1000 coral reefs MPAs in 2007, when the surveys were conducted [22, 69]. Seventy-eight responses were received from 33 countries, hence the sample population was approximately 7% of the total population. The sample population was heterogeneous (see S3 Table for sample statistics), with 15% of MPAs situated in Africa, 46% the Americas, 30% Asia and 9% in the Pacific. In terms of IUCN category, 16% of MPAs were category I or II, 16% III or IV, 25% V or VI and 42% unset. Since expert scoring can be inaccurate, prone to subjective perceptions and strategic responses [62, 70], academics and NGO employees were included as well as management staff. Expert respondents comprised 34% management staff, 33% scientists, 28% NGO staff and 5% government staff (self reported and based on their principal employment).

Given that self selection can generate sampling biases, it is important to assess the representativeness of our sample, so we tested to see if there were significant differences in terms of IUCN category (I to VI and unset), as well as region between our sample and the MPAs included in the reefbase database [69]. There were no significant differences (X^2^ = 2.7, n = 65, P = 0.85 and X^2^ = 3.35, n = 66, P = 0.85 respectively). Status as a developing country was based on the reefbase category. In our sample 80% of MPAs were situated in less developed countries, compared to 82% of the reefbase database MPAs. Median budgets per km^2^ were almost double those reported by Balmford et al., [59], but the percentage with no budget was the same. 11 MPAs had multiple surveys submitted, for which less than 10% of responses differed (apart from the Great Barrier Reef Marine Park and Komodo National Park, where 11% and 17% of responses varied respectively). One survey was included at random from duplicated sites, so that 66 MPAs were included in the final dataset.

### Data analysis

The number of informative performance variables was reduced to 13 from an initial pool of 27, by prioritising those with spatial or temporal comparisons [38, 41], those demonstrating adequate variation and those not highly correlated with one another (Table 1, S1 Table). A correlation matrix was generated to examine the direction and strength of association between these 13 performance measures (Table 2), using Spearman rank correlations, as in [71].

**Table 1:** MPA performance measures for ecological, social and economic outcomes, as well as threats, achievement of goals and perceived success. See S1 Table for detail on coding.

Outcome type	Performance measures	Min	Max	Mean	Median	SD
**Ecological**	**Change in live coral cover since established**	**-34%**	**33%**	**-0.23**	**0.2**	**12.2**
	**Live coral cover compared to country average**	**-23%**	**77%**	**7.6**	**1.75**	**23.2**
	**Perceived changes in fisheries**	**-1**	**1**	**0.55**	**1**	**0.67**
	**Perceived changes in species conservation**	**-1**	**1**	**0.69**	**1**	**0.53**
**Social**	**Perceived change in stakeholder conflict**	**-1**	**1**	**0.26**	**0.5**	**0.8**
**Economic**	**Perceived greater wealth for local communities as a result of MPA**	**0**	**1**	**0.46**	**0**	**0.5**
	**Estimated Number jobs supported per km^2^ managed**	**0**	**2460**	**123**	**2.2**	**443**
**Threats**	**Number of destructive activities that have decreased inside the MPA over time**	**0**	**9**	**2.5**	**2**	**2.3**
	**Difference between number of large scale threats inside and outside MPA**	**-8**	**4**	**0.03**	**0**	**1.9**
	**Number of destructive activities staying the same / decreasing inside, but not outside MPA**	**0**	**8**	**1.4**	**1**	**1.8**
**Goals**	**Number of banned activities occurring**	**0**	**10**	**2.7**	**2**	**1.9**
	**Perceived extent of primary aim achieved**	**0**	**3**	**2.7**	**2**	**1.9**
	**Perceived success of the MPA in general**	**0**	**3**	**1.9**	**2**	**1**

**Table 2:** Spearman rank correlation coefficient matrix for success variables. N = 66. Only variables with p values < 0.01 are reported.

	Achievement of primary aim	Temporal coral cover change	Spatial coral cover comparison	Change in fisheries	Change in species conservation	Diff in threats inside / outside	No. destructive activities decreasing	No. destructive activities decreasing compared to outside	No. banned activities occurring	Change in conflict	Increased wealth	Increased employment	Total jobs supported / km^2^
**Overall success**	0.784***		0.312**	0.641***	0.663***				-0.277 **		0.493**	0.406	
**Achievement of primary aim**	-	0.262*	0.334	0.548	0.622				-0.247*		0.493***	0.392**	
**Temporal coral cover change**	0.262*	-	0.473***			0.392**	0.288**						
**Spatial coral cover comparison**	0.334 **	0.473 ***	-		0.219 *	0.212 *					0.248 *	0.247 *	
**Change in fisheries**				-	0.582 ***	0.233*					0.451 ***	0.331 ***	0.227 *
**Change in species conservation**	0.663 ***		0.219 *	0.582 ***	-				-0.239 *		0.297 **	0.260 **	0.227 *
**Diff in threats inside / outside**		0.392 ***	0.212 *	0.244 *		-					0.219 *		
**No. destructive activities decreasing**		0.289 **					-	0.399 ***	0.405 ***				
**No. destructive activities decreasing compared to outside**							0.399 ***	-	0.293 **				
**No. banned activities occurring**	-0.247 *				-0.239 *		0.405 ***	0.293 **	-				
**Change in conflict**										-			
**Increased wealth**	0.493 ***		0.248 *	0.451 ***	0.297 **	0.219*					-	0.711 ***	
**Increased employment**	0.392 **		0.247 *	0.331 ***	0.260 **						0.711 ***	-	

* = p<0.1

** = p<0.05

*** = p<0.001.

We used the existing literature to develop hypotheses as to the many variables that would be expected to affect MPA performance outcomes, here-after referred to as “drivers” of performance. The expected direction of influence of each of the 42 drivers was based on previous research (Table 3, S8 Table). Because of the multidimensional nature of performance, some performance measures were also drivers of other performance measures (e.g. the number of banned activities taking place within the MPA was both a social performance measure and a potential driver of ecological performance). Drivers that can be influenced by management were defined as endogenous and those over which management has no control, as exogenous. The impact evaluation literatures refers to these as “treatments” and “moderators” (contexts) respectively [41, 72]. Drivers were categorised into groups, including MPA attributes (such as size and features); funding, specific aims of management and management actions (such as education and monitoring). Exogenous variables included the regional location, local and national contextual variables and large scale threats. Non-linear, interacting and dynamic relationships were expected for several variables, for example ecological improvements can lag behind socio-economic benefits [50, 73], while costs can occur immediately, potentially reducing support and compliance [5, 49]. MPA size may also have a non-linear relationship with performance, with either small or large MPAs achieving the best outcomes [74]. Such relationships are poorly understood.

**Table 3:** Comparison of significant predictors of MPA performance observed in this study against hypotheses generated from previous research. Italics denote exogenous variables. See S8 Table for reference list of research used to generate hypotheses.

	Aspect	Expected ^a^	Observed ^b^
MPA features	MPA size	NL, +	+ +
	Existence or size of no-take area	+	+ ++--
	Age	NL, +	+-NL
	Low IUCN number (strict regulations)	+	-
	Zoning	+	+ ++
	Community managed	+	+
	Government managed	-	NS
	Multiple (co) management	+	-
	Part of physical or monitoring network	+	NS
Aims	Multiple aims	-	- -
Management actions	Management plan exists	+	+ +
	No. staff	+	+ +
	Staff training	+	NS
	No. regulations or bans on destructive activities	+	++ -
	% activities detected and/or enforced	+	+ + + +
	Community consultation	+	?
	Community participation, institutions	+	+ +
	Community incentives, alt. livelihoods	+	+ + +
	Environmental education and outreach	+	NS
	Conflict resolution mechanisms	+	NS
	Social and ecological monitoring	+	-
	Management effectiveness evaluation	+	NS
	Technical supervision from outside organisation	+	NS
	Compensation to groups suffering costs	+	+ +
Financial	MPA funding (absolute / per area / for active management costs)	+	+ + NL
	Facilities, equipment and infrastructure	+	?
	% funding from user fees	+, -	NS
	% funding to local community projects	+	NS
*Threats / uses*	*No*. *threats*	*-*	*----*
	*Number of fishers / fishing pressure*	*-*	*+--*
	*Number of visitors/ visitor pressure*	*+*, *-*	*+*
*Local / national context*	*Increased tourism*	*+*, *-*	*+*
	*Coastal zone management beyond MPA*	*+*	*+ -*
	*Fisheries management*	*+*	*+ +*
	*Less developed country (LDC) / GDP pc*	*-*	*+*
	*Human development index (HDI)*	*+*, *NL*	*+*
	*% reefs at risk*	*+*, *-*	*+ +--*
*Region* ^*c*^	*Asia*	*-*	*+ + +*
	*Americas*	*-*	*-*
	*Pacific*	*+*	*+*
Survey variables	Respondent member of management staff	+	NS
	Expert estimate for percentage coral cover (not based on survey data)	+	NS

a. Hypothesized direction of endogenous and exogenous variables on performance; positive (+), negative (-) or non-linear (NL).

b. The number of symbols indicates the number of times a significant relationship was demonstrated in the 8 performance regressions.

NS denotes that no relationship was detected.

Performance measures and endogenous explanatory variable values were generated principally directly or indirectly from survey responses and exogenous variables were supplemented with data from public sources (see S1 and S2 Tables for detail and S1 Dataset for the raw data with sensitive data including respondent identity, performance measures and financial information removed). Almost all respondents reported single values for performance measures, but where they reported a range, the median value was used in the regression analysis. Variables that were not included due to lack of respondent awareness or accurate indicators, but which might be expected to have an impact on performance, include biophysical aspects of habitats or fish populations and socio-economic and cultural factors [16, 19, 30, 75]. Several variables could only be incorporated into the analysis using weakly correlated variables or national datasets due to lack of respondent knowledge. For example, local human population density was included using data on visitor and fishing pressure and employment data or national level data.

Eight of the 13 measures were chosen that represented distinct types of performance and lacked strong correlation with the other performance measures. These were; the perceived extent that primary aim was achieved, the perceived success of the MPA in general, the change in live coral cover since establishment, perceived changes in fisheries health and in local community wealth, the number of destructive activities that have decreased inside the MPA over time and the difference between number of large scale threats inside and outside MPA. Bi-variate analyses are of limited use in exploring causal relationships and are subject to spurious associations [75]. Multiple regression can incorporate interactions and confounding effects between inputs, outcomes and contextual factors [16, 30, 54], since these effects can obscure or alter the impact of endogenous variables on outcomes [38, 41, 72]. The large number of explanatory variables that have been shown in the literature to influence performance, together with missing variables and small sample sizes, meant that starting with full models and using information theoretic approaches to reach a minimal adequate model was not appropriate for this dataset.

Therefore, multiple regressions were conducted to test the following underlying model for each of the eight measures of performance (P):

P=f(At,Mng,Fin,Thr,Ctx,Nt,Sv)+e (1)

where P = performance, At = MPA attributes, Mng = management actions, Fin = financial aspects, Thr = local threats, Ctx = local context, Nt = national context, Sv = survey variables, e = error.

Drivers of each of the eight measures of performance were tested using separate regression analyses. This was critical as combining distinct types of performance into composite scores masks underlying relationships such as differing drivers for each outcome. Additionally certain outcomes, such as conflict reduction, may not constitute an aim at every site, hence may not always be relevant to performance. Finally, measures can be both outcomes in themselves and drivers of other outcomes (for example conflict, increased tourism or ecological decline).

The distribution of the performance indicator data determined the type of regression analysis used. Ordinary least squares regression was used for normally distributed and continuous data e.g. coral cover comparisons. If Shapiro-Wilks tests for normality were failed, data were log-transformed and then regressed using ordinary least squares. Logistic regression was used for binomial data. Ordinal variables were explored with ordered logit regressions. A negative binomial regression was used for count data, e.g. for the number of large- scale threats inside compared to outside. Quadratic functional forms were used for several variables for which there was an a priori expectation of nonlinear effects based on the literature, such as MPA age, size, no-take area size and budgets. In addition, interactions were explored between variables with an a priori likelihood of being inter-related e.g. MPA age and size (we would expect MPAs which are both large and old to have more positive outcomes than MPAs which are either large or old [30]), the number of staff and the MPA budget, and tourist and fishing pressure. Survey variables representing potential sources of bias, including respondent affiliation [76, 77] and data quality, were also included.

Initially, models with a few potential explanatory variables (age, size, region, no-take area, co-management, part of network, ecological monitoring and budget) were developed, since these variables had the strongest evidence that they affected MPA performance based on the literature review (S8 Table). Correlations were assessed between the dependent variables and between the explanatory variables and any variables that were significantly correlated were excluded. Given the very large number of potential interactions, and therefore the risk of Type II errors, we focused our investigations on variables that had a priori support from the literature, so as to test specific hypotheses (Table 3). Certain variables with strong evidence of impacts on many types of performance were tested in each model. However, other variables were tested based on their hypothesized effects generated by the literature review, on specific types of performance, including management actions, threats, uses and contextual information. The order in which the variables added was based on the strength of evidence in the literature review that they affect each type of performance (S8 Table). Variables that were non-significant (p > 0.1) were removed and another variable added using a stepwise procedure, required due to the low degrees of freedom and large number of potential variables to test. Variables were kept in the model if they had sufficient support. Successive models were compared against each other using analysis of variance. This process was repeated, until a single final minimal acceptable model (MAM) was reached for each measure, where removal or addition of any variable did not change model fit significantly. Model assumptions were tested at each step, including normally distributed errors and homoscedasticity. Each final model passed post-hoc tests for model fit and normality of residuals.

## Results

### Relationships between individual performance measures

We obtained a constrained set of performance measures covering ecological, social and economic outcomes (Table 1). On the ecological dimension, MPA coral cover was reported to be higher than the mean coral cover for each country, and on average no change in coral cover had occurred since MPA establishment. Respondents perceived improvements in species conservation in 72% of MPAs and fisheries improvements in 66%. In terms of local welfare changes, half the MPAs were reported to have increased local wealth, while stakeholder conflict was perceived to have decreased in only a quarter of MPAs. Banned activities were reported to occur in 80% of MPAs. The data on employment were highly skewed by a few MPAs with many associated businesses, but the median was 2.2 jobs supported per km^2^ protected. When evaluating overall performance, respondents were more likely to say that the MPA had been successful (32%) than that it had achieved its primary aim (11%). Generally, spatial and temporal comparisons were highly variable, with improved performance for some and not other ecological, socio-economic, threat and goal based measures.

Results given by different respondents for the same MPA had a high level of congruence, suggesting reasonable accuracy of reporting [57]. Similarly, responses to differently worded questions demonstrated the internal validity of the survey. For example, reported changes in coral cover from monitoring and one-off studies were highly correlated with perceived changes in habitat quality (F = 3.41, df = 2, p = 0.041), while MPAs with perceived improvements in habitat quality had a mean change of +2.9% in live coral cover compared to those with no perceived improvement, which had a mean of -6.8%. Anecdotal evidence of spill-over was highly correlated with perceived improvements in fisheries (chi^2^ = 11.0, n = 60, df = 2, p = 0.027).

**Fig 1:**
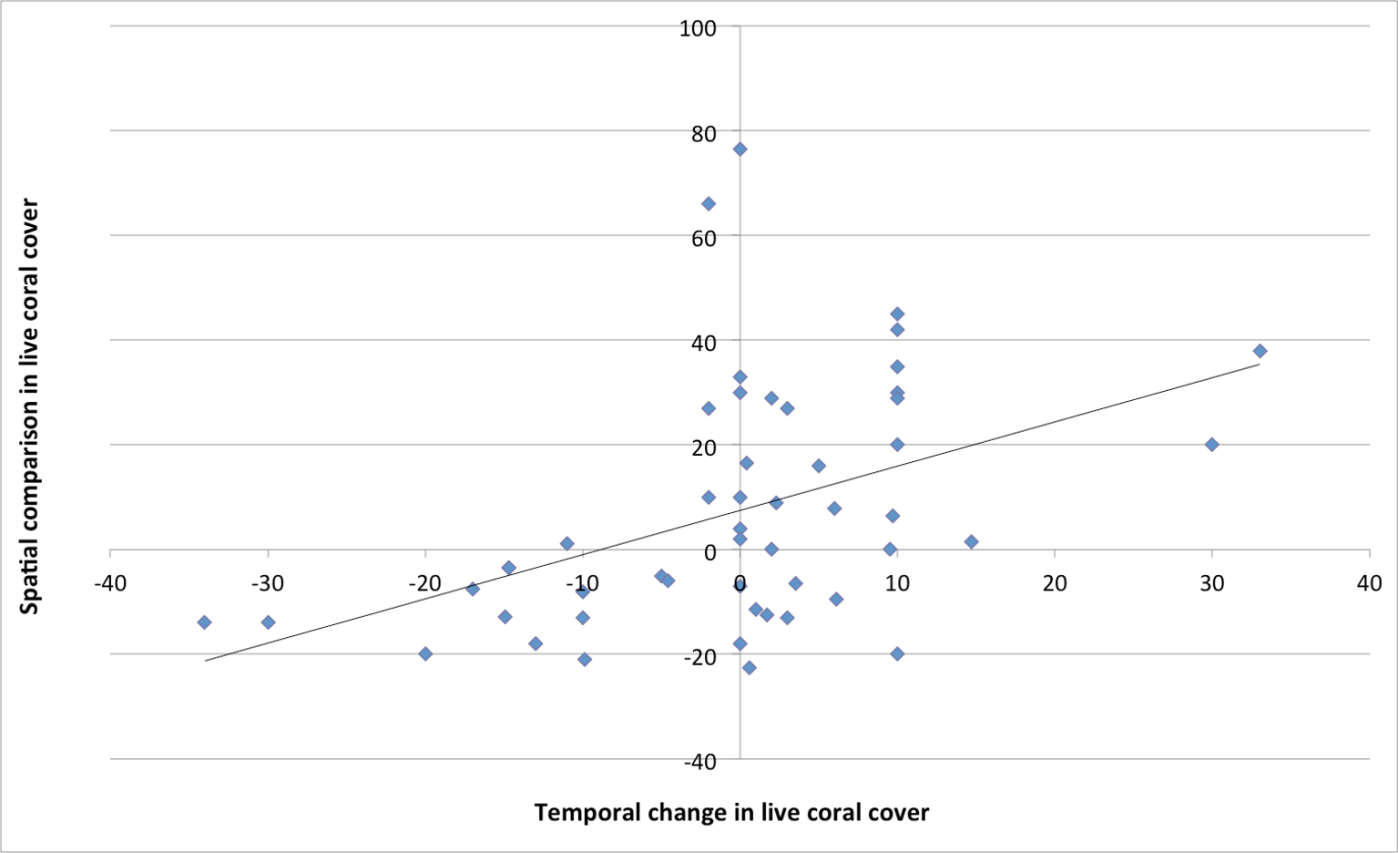
The relationship between spatial and temporal live coral cover comparisons (f = 12.2, n = 50, df = 1, p = 0.001, R^2^ = 0.2).

Correlations between different measures of performance were used to elucidate relationships between them (Table 2). Certain improvements were highly correlated. Habitat quality, fisheries and species conservation improvements were correlated with increased wealth and employment. Perceptions of general success and of whether the MPA’s primary aim had been achieved were highly correlated (Spearman’s Rho = 0.784, n = 66, p = 0.000). Habitat improvements were correlated with the achievement of the primary aim but not with perceived overall success. Neither success nor achievement of the overall aim correlated with changes in threats, destructive activities, conflict reduction or job creation. Instead they were linked to higher coral cover inside than outside the MPA, species and fisheries improvements, compliance and economic benefits. Perceived improvements in coral cover over time were correlated with reduced threats inside the MPA compared to outside, as well as reductions in the number of destructive activities. Good coral cover in comparison to the national average was linked to improvements in species conservation, reduced threats and economic improvements. Improved jobs and employment were correlated with both endangered species and fisheries improvements. Interestingly, change in conflict was not correlated with any other outcome. Spatial comparisons in coral cover (between the MPA and the surrounding areas) were highly variable and explained only 20% of the variation in coral cover change over time within the MPA (Fig 1).

### Drivers of MPA performance

It was less likely that an MPA was perceived to have achieved its aims if it had multiple aims, but more likely if the principal aim related to increased tourism (see Table 4 for regression summary and S4–S7 Tables for full regressions). Having a greater number of zones, more staff, more regulations, fewer internal threats, benefit sharing and development initiatives all contributed to aim achievement. MPAs in countries with lower per capita GDP and those in nations where a high percentage of the reefs were threatened had met their aims less, as expected (S4 Table). Perceptions of MPA success were largely associated with different variables from aim achievement, apart from more zones, higher GDP and fewer reefs at risk nationally. Asian MPAs were perceived to have had greater success than those elsewhere. Enforcement, community associations, greater funding, of which a higher portion was retained, also contributed to perceived success.

**Table 4:** Significant variables related to MPA performance, summarising the final multivariate models (in S4–S7 Tables) for each of 8 key performance measures, organized by type of input. + denotes a positive co-efficient,—a negative co-efficient. The number of symbols denotes the p-value (i.e. + = p<0.1, ++ = p<0.05, +++ = p<0.01). Exogenous variables are denoted in italics.

Performance measure (N)	Achievement of Primary Aim (60)	Perceived MPA success (46)	Increase in wealth (40)	Conflict has decreased (59)	Temporal change in coral cover (57)	Improvement in fisheries (48)	No destructive activities decreasing (49)	No. threats compared to outside (39)
**MPA features**	No. zones +++	No. zones ++	No take area -	Size no-take ++	No. zones ++	No-take area—-	Age +++	Age^2^ -—-
	Age	No take area—-	Low IUCN category -	Community managed +	No. staff—- -		Size (km2) ++	Size no-take +++
	Size (km2) ++				Age—- -		Age * size—- -	
	Age * size—-				Multiple mngt-—-		Size no-take +++	
							Mooring buoys +++	
**Aims**	Tourism aim ++		Multiple aims -					
	Multiple aims—- -							
**Management actions**	Staff per km^2^ +++	% illegal activities punished ++	No activities banned -	Alternative livelihood project +	Management plan +++	Compensation+	Frequent research / monitoring—- -	% illegal activities punished ++
	Benefit sharing project(s) +++	Community institution(s) ++	Management plan +		Fisher compensation +++	% illegal activities detected+		Staff per km^2^ +++
	Development initiative(s) +++		% illegal activities detected ++			Community institutions +		
	No. banned activities +++					No regulated activities +		
**Financial**		% funding returned to government—-		% funds from intl. organizations ++	% funds used for management costs ++		International conservation grant +++	(Funding per km^2^)^2^ -—-
		% funding from donations ++						% funds used for management costs +++
		Funding per km^2^ +						
**Threats / uses**	No. threats inside—-			No. threats inside—-	No. threats inside—- -	No. threats inside—-	Rank commercial fishing ++	Rank commercial fishing—-
					Rank subsistence fishing—-			
**Local context**			*Fisheries management +*			*Increased tourism +*		*Fisheries management +++*
**National context**	*GDP pc ppp +++*	*GDP pc ppp +*	*LDC ++*	*% reefs high risk ++*	*Coastal zone management + +*	*Human development index +*	*% Reefs at risk +++*	*Coastal zone management*—*-*
	*% reefs high risk—- -*	*% reefs high risk -*						
**Region**		*Asia ++*	*Pacific +*		*Asia +++*	*Americas -*	*Asia +++*	
**Survey variables**								NGO employee +++

Coral cover improvements were observed in MPAs with fewer internal threats, formal management plans, more zones, higher funding for active management and compensation for fishers and as well as those located in areas with lower prevalence of subsistence fishing, with coastal management or in Asia (Table 4, S5 Table). Older MPAs were reported as having suffered greater coral cover losses. Fisheries improvements had been observed in MPAs with more regulations, greater detection of infractions, affiliated community institutions, those making compensation payments, having fewer threats inside and increases in employment or tourism, and located in areas with a higher human development index. MPAs in the Americas reported greater fisheries declines.

Sites with fewer restrictions or banned activities and smaller no-take areas were perceived to have increased local wealth more, which may be because they had limited extraction less (Table 4, S6 Table). MPAs with formal management plans and higher infraction detection rates, located in developing countries, the Pacific or an area with fisheries management had overseen greater increases in wealth. Greater conflict reduction was reported at sites located in countries with highly threatened reefs, with community management, substantial international funding, alternative livelihood schemes and fewer internal threats.

Destructive activities had decreased more in older, larger MPAs, with larger no-take areas, with mooring buoys, less commercial fisheries activity and large international grants (Table 4, S7 Table). MPAs situated in developing countries, those in Asia or in countries with a greater proportion of reefs at risk had decreased damaging activities more. MPAs with fewer threats inside compared to outside were better staffed, spent more funds on active management, punished a greater proportion of illegal activities, with large no-take areas, active fisheries management and limited commercial fishing activity. Coastal zone management reduced the difference between the number of threats inside and outside an MPA's boundaries. Budget per area showed a non-linear relationship to threats, which could be due to returns to scale. NGO staff were more likely to report threat reduction than other respondents.

### A comparison of drivers between performance measures

For the final models, sample sizes ranged from 39 to 60 and adjusted R^2^ ranged from 0.67 to 0.84 (see S4–S7 Tables for model parameters). Models that included financial variables had smaller sample sizes, as only 40 responses provided full financial data. Generally there was marked variation between performance measures in which explanatory variables were significantly associated with them, even between measures within the same broad category (Table 4). However MPA features such as size, age and presence of no take areas were important explanatory variables for all measures of performance. MPA age had a non-linear relationship to eliminating threats inside and there was an interaction between age and size for aim achievement and reducing destructive activities, so that the lowest reduction in destructive activities was felt in MPAs of intermediate size and age. The number of threats occurring inside the MPA, including fishing pressure and reefs at risk, was a highly significant driver for almost all performance measures.

Management actions such as active fisheries management, staffing, enforcement and benefit sharing were generally important, although less so for reduction in conflict or destructive activities. Financial variables emerged as important for several types of performance, such as threat reduction and perceived success, relating not to absolute levels of funding but to sources of finance e.g. proportion funds from donations or international organisations and to spending specifically on on-site management activity rather than returning to general governmental budgets. Funding capacity has been found to be critical for fisheries performance [32], but it is correlated with other management inputs which we included directly in our analysis.

National contextual variables relating to economic development, coastal zone management and the percentage of reefs at risk were more significant than local contextual factors, such as local economic development or cultural erosion. MPAs in more developed countries were seen as having more success both generally and in the context of fisheries improvements. However several drivers demonstrated the additionality achieved by management, including greater wealth increases for MPAs in less developed countries, greater threat reduction in areas with higher percentage of reefs at risk or conversely less threat reduction in locations with wider coastal zone management. Similarly, perhaps because Asian reefs are particularly highly threatened, coral cover improvements and reductions in destructive activity were more marked in MPAs there. Overall, endogenous factors explained a greater proportion of performance variation than exogenous ones.

Adjusted R2 values of at least 0.67 suggest that the drivers included in the multivariate analysis explained a large proportion of the variation in each performance measure. However, stepwise model selection procedures have been known to produce inflated R2 values [78], so these results should be treated with caution. A survey design variable was only a significant predictor once, and only for one performance measure.

Several variables were significant in the direction expected from the literature (Table 3), including zoning, extent of regulation, fisheries management, the level of enforcement and threats occurring, community management or institutions and incentive initiatives. Several variables also showed different results for different performance measures; the existence or size of a no-take area which enhanced three and reduced two types of performance, MPA age which showed positive, negative and non-linear effects, and the number of banned activities which reduced wealth enhancement but contributed to aim achievement and fisheries improvements. Similarly, the local reliance on fishing, wider coastal management and the percentage of reefs at risk enhanced some types of performance and reduced others. Those variables that were significant but in an unexpected direction included co-management (reducing coral cover gains), research and monitoring effort (reducing the abatement of destructive activities), no-take areas (decreasing the likelihood of fisheries improvements) and larger no-take areas (decreasing conflict). Variables which unexpectedly did not emerge as significant included the type of management organisation, the set-up budget, the absolute level of staffing or funding, visitation rates, on-site revenue raising, staff training, outside technical assistance, education programs, fisher pressure and being part of a physical or monitoring network.

## Discussion

While performance between MPA sites was highly variable with improvements in certain ecological and socio-economic outcomes were frequently coupled, as has been observed elsewhere [10, 11]. For example habitat quality, fisheries and species conservation improvements were associated with increased wealth and employment. Reduced threats were also coupled with habitat improvements. Unlike other measures, conflict reduction was not correlated with other types of performance (as has been observed in the Caribbean [17]) and the regression model had the lowest number of explanatory variables. This is not unexpected as complex social processes are unlikely to be adequately investigated using this quantitative approach, so that relevant socio-cultural factors are likely to be missing from the analysis. Since several measures of performance were not coupled, our analysis suggests that aiming for high levels of performance against multiple goals is potentially unrealistic, as trade-offs are unavoidable [1, 16, 79]. Indeed having multiple aims was a significant predictor of lack of aim achievement.

Respondents distinguished between general success and the achievement of the MPA's primary aims. These two measures were also influenced by different explanatory variables. This highlights the fact that perceived performance incorporates distinct, sometimes contradictory goals and multiple dimensions that are not well represented by single composite scores [31, 33]. Our results were consistent with the widespread reporting of MPAs increasing tourism and related employment [80] as well as maintenance (rather than improvement) of coral cover inside the MPA compared to outside [20]. Our analysis supports previous findings that socio-economic factors are important drivers of a number of performance measures [51, 56], such as improvements in coral cover, fisheries and conflict. However the perception of socio-economic versus ecological drivers is perhaps a false dichotomy, since such factors are so inter-related [8, 9].

Each MPA is unique in terms of the economic, social, political and institutional context in which it operates, at community, national and international scales, which makes it challenging to transfer lessons between MPAs [6]. However, a global analysis can give a broad understanding of the likely drivers of coral reef MPA performance, by including a wide range of endogenous and exogenous explanatory variables. By analysing different measures of performance individually, we acknowledged different perceptions of what constitutes performance, the multiplicity of MPA goals, the ability of MPAs to achieve some positive outcomes without meeting others and the confounding interactions between variables. This is the first time that a multivariate analysis has been carried out to model drivers of distinct types of performance in coral reef MPAs globally, using a single dataset and methodology. Although no quantitative analysis can fully characterise such complex social and ecological systems and include all potential drivers, our approach is an advance in that it uses outcome scoring which is not simply binary, but instead spatial and temporal comparisons and multivariate statistics, allowing multiple factors and interactions to be included to better represent MPA performance [30, 41]. Demonstrating causal relationships based on observational data is challenging [72, 75], however it is possible to infer that such relationships may exist based on the explanatory power of input variables, and their consistency, plausibility and congruence with previous research [81].

Different measures of performance had different drivers [9, 20, 82]. This means that MPA features or management actions that enhance certain types of performance may reduce other types, similarly to what was observed for co-managed fisheries [11]. For example, the ideal number of zones or the size of the no-take area appears to depend on whether habitat, fisheries or socio-economic improvements are prioritised. This also means that composite measures of performance can mask relationships between different outcomes and between specific outcomes and their drivers. Each site needs to prioritise their goals and make trade-offs between incompatible aims, so that the most desired site-specific outcomes of management can be achieved using targeted inputs for that aim [6, 14, 83]. Co-management did not increase performance and conflict had only decreased in 24% of the MPAs in the study. Further research should explore if this is due to new incentives for extraction, inequitable benefits or challenges associated with co-management related to bureaucracy, local capacity and funding uncertainty [9, 55, 84] or due to a lack of consensus, or contradictory aims. Alternative livelihoods schemes had resulted in decreased conflict, but were not significant in other outcomes, perhaps as they frequently fail to generate livelihoods linked to conservation outcomes [85, 86].

MPAs showing the greatest improvements over time in coral cover, fisheries, conflict and threat reduction and achievement of primary aim were located in areas where there were few pre-existing threats (including intensive fishing) and where there was coastal zone management [21, 53, 87]. That the number of threats inside the MPA is such a strong driver of multiple types of performance (as expected [57, 88]), highlights the need for careful consideration of the existence of multiple threats when locating new MPAs, as threats may act cumulatively. Nevertheless, endogenous factors were more important than exogenous ones in determining MPA performance; MPA design features (e.g. adequate sizing) and management (e.g. enforcement) were more important in determining performance than local and national contextual factors (e.g. extent of poverty, unemployment or population growth) [11, 31, 52], which are beyond the control of management. Performance did not seem to be highly dependent on contextual factors, contrary to what has been hypothesized [54, 57]. This is encouraging since it means managers have the power to enhance performance despite a challenging external environment and that certain features and actions can generally improve MPA outcomes. The effects of MPA age and size on performance in this study were complex, non-linear and interacted with one another, as has been suggested previously [20, 44]. Factors which would ideally be included in future analyses of MPAs, but which could not be addressed here, include community heterogeneity, incorporation of traditional management structures, strong leadership, property rights aspects and perceived equity or transparency of enforcement or benefit provision [8, 16, 56, 81]. While variables indirectly related to local population density such as number of business and people employed did not emerge as significant predictors of performance, future research should seek to incorporate griddled population datasets to test for the impact of population density more directly, as well as improved ecological datasets and finer resolution contextual data.

Our finding that for our sample, research, training and education are of less importance in driving performance than community-focused and institution-building activities is contrary to other research that stresses the importance of education [1, 89] and frequent research and monitoring [33, 90]. This is not to say that these factors will not increase performance at certain sites or once other critical inputs, such as fisheries management and enforcement, are present. However the results do highlight the crucial role of local community engagement, benefit provision or compensation in driving several types of performance, as has been reported previously [6, 17, 24, 33]. Since so much emphasis is placed on the need for MPA networks [83, 91], further research is needed to clarify why physical or monitoring networks were not significant predictors of any type of performance. This may be due to differences in institutional capacity or co-ordination between MPAs [92, 93] or due to time lags in network emergent properties [83].

Research has suggested that MPAs in less economically developed countries and are less effective [31, 33, 94] and reefs in Asia are more threatened [95, 96]. However our results suggest that, within our sample, MPAs in Asia and developing countries may be more effective than those in developed countries, in terms of the improvements they generate over time and compared to unmanaged areas, all else being equal.

Our approach is subject to important limitations. Our assumption that respondent evaluations are valid and broadly comparable does not account for likely differences between the extent of respondent knowledge and that self-selected respondents may have strategic reasons or personal biases that distort their responses and sample MPAs are likely to be better funded and more successful than MPAs generally. The sample was comparable with the global population in terms of regulations and regional location and included many MPAs without active funding. Additionally, the diverse affiliations of respondents should have reduce respondent bias [31, 97]. However the use of a single respondent per site remains a critical limitation due to the potential for subjectivity and it is not possible to test the extent to which self-section bias has influenced the reported performance for each MPA or the extent to which this dataset is representative in terms of MPA performance globally. Future applications of this methodology could address this through increasing the MPA sample size or using a randomised sample and through triangulation of multiple responses from respondents with different affiliations. Here, larger sample size was prioritised over a randomised sample of respondents, with the use of tests for representativeness and affiliation-related bias to give confidence in the results. Small sample sizes and clear a priori hypotheses made a stepwise approach suitable for this analysis, however, future work to increase sample sizes would also allow use of other model selection procedures, such as information theoretic approaches, which would improve the robustness of the results, particularly in terms of model fit [78].

We have provided conclusions in terms of MPAs generally, but it is likely that MPAs in temperate habitats would have very different ecological dynamics, so our inferences apply only to coral reef MPAs. The approach used here can explore correlations between drivers and different measures of performance. In order to demonstrate causation between MPAs inputs and ecological and socio-economic outcomes, we would need to undertake before-after-control-intervention trials to control for the effect of confounding variables [41, 98]. Such an approach has been undertaken for five socio-economic indicators related to poverty in eight villages in Indonesia [99]. This approach is critical to demonstrate causation of interventions, but is not feasible at large scales. Performance measures would provide more robust information if they combined spatial and temporal comparisons. Spatial comparisons are subject to bias if the MPA was designated as it contained higher quality habitats or higher visitation levels than nearby areas. This could explain the weak correlation between temporal and spatial cover measures. Combining spatial and temporal comparisons would help to elucidate if changes inside MPAs are different from those outside given location biases. Only one of our 13 measures included such a comparison; the number of destructive activities staying the same or decreasing inside the MPA, but not outside. Future work should seek to develop combined indicators to increase our level of confidence in performance indicators.

Our approach is intended to complement rather than replace research utilising fine scale ecological or socio-economic data, stakeholder perceptions and in-depth qualitative information collected on-site. Using expert opinion to gather information on a range of performance measures was cost effective and allowed for spatial and temporal comparisons. It could be used as part of an adaptive management approach to track changes in MPA performance globally at minimal expense, ideally with multiple responses for larger numbers of MPAs and repeated over time as contextual and management factors change [23, 52, 100]. Although detailed site-specific evaluations of the drivers of MPA effectiveness are required to generate concrete site-specific management advice, this global-scale quantitative approach is critical in testing hypotheses concerning drivers of performance, highlighting the broad components and correlates of specific types of performance and so providing support for decisions as to efficient use of limited funds and effort and promoting accountability within an adaptive management framework.

## Supporting information

S1 Text Survey instrument for data collection. (DOCX)

S1 Table Coding of the performance measures. (DOCX)

S2 Table Source and units for explanatory variables used in regressions. (DOCX)

S3 Table Sample population statistics (N = 66 unless otherwise stated). (DOCX)

S4 Table Results of ordered logit regressions to determine significant variables related to achievement of MPAs aims and overall success. Standardised coefficients are given, together with model summaries. For explanation of the explanatory variables see S4 Table. * = p<0.1, ** = p<0.05, *** = p<0.001.
(DOCX)

S5 Table Regression to determine significant variables related to temporal changes in live coral cover (ordinary least squares) and improvements in fisheries (logistic). * = p<0.1, ** = p<0.05, *** = p<0.001.
(DOCX)

S6 Table Logit regressions to determine significant variables related to increase in wealth and decrease in conflict at MPAs. * = p<0.1, ** = p<0.05, *** = p<0.001.
(DOCX)

S7 Table Ordinary least squares regressions to determine significant variables related to the natural log of the number of destructive activities decreasing over time and the number of large scale threats inside the MPA compared to outside. * = p<0.1, ** = p<0.05, *** = p<0.001.
(DOCX)

S8 Table Variables hypothesized to impact facets of MPA success. This table summarises the variables used in this study that been shown quantitatively or anecdotally to influence MPA performance in the literature. For expected direction of the impact on performance, NL denotes a non-linear relationship, + a positive and–a negative. References are listed below.
(DOCX)

S1 Dataset Raw data from management survey. Sensitive information relating to respondent identify, financial information and performance indicators has been removed.
(XLSX)
