# Supplementary material for: Drivers of coral reef marine protected area performance
Source: PLoS One. 2017 Jun 23;12(6):e0179394. doi: 10.1371/journal.pone.0179394 (PMC5482435; doi:10.1371/journal.pone.0179394)
Supplement: S1 Text — (DOCX) [file pone.0179394.s001.docx]

**S1 Text. Survey instrument for data collection**

**Introduction.** Thank you for participating in this survey about marine managed areas (MMAs). We are targeting MMA managers. We are also interested in receiving replies from researchers with a good current working knowledge of an MMA, based on at least a year’s involvement with it. The MMA should contain at least 50% marine habitat and an area of coral reefs. If you fit this profile, we would be very grateful if you could answer the questions below. We would like to look at trends from many MMAs around the world, so your answers are very important to us.

This questionnaire is part of an on-going research project by Venetia Hargreaves-Allen, from Imperial College London, with support from the World Fish Centre and Reefbase. We are hoping to increase understanding of MMA management by benefiting from your experience. We are researching costs and benefits generated by MMAs and how these are linked to conservation success. This will enable us to make recommendations, including identifying strategies for enabling more sustainable support for MMA management costs. Please be aware that all your responses as strictly confidential and will not be linked to your specific MMA. Summary results only will be published. If you would like a copy of the report produced from this study, please add your email at the end of the questionnaire.

**Personal Details.**

Your name­­­­­­­­­­­­____________________________________________________________________________________________________________________

Name and country of protected area________________________________________________________________________________________________

Your position (please give as much detail as possible):__________________________________________________________________________________

Contact e-mail address ______________________________________Telephone number_____________________________________________________

Has there been any scientific or social research done at your MMA? If yes, please give details of any reports or publications and if possible, please list them): ______________________________________________________________________________________________________________________________

______________________________________________________________________________________________________________________________

Enter the address of any website(s) that contain information on this MMA:_________________________________________________________________

**Background Information.**

1. Total marine managed area (MMA) size *___________________________* km^2^
2. Year of formal designation _____________________ Not applicable □
3. Please list all designations including IUCN category, national and international designations e.g. World Heritage site: _________________________________________________________________________________________________________________________

4) Which best describes the management structure at your MMA (please choose one)?

Local or central government department or government agency □

Community-based management □

Government and local community co-management □

Government and NGO co-management □

Other (please specify) ____________________________________________ □

1. How many zones (if any) are there in your MMA? If no zoning exists, please mark 0.

0 □ 1 □ 2 □ 3 □ 4 □ >5 □

6) Is there an area or zone where no human uses are allowed (except research)? Yes □ No □ Not sure □

If yes, please state size ____________ km^2^

7) What percentage of live coral cover is currently found, on average in the following areas? ** Please give details, including dates of any publications at the beginning of the questionnaire.*

(a) within your MMA, when it was established? ________________ % Don’t know □

How have you estimated this? Expert judgement □ On-going scientific monitoring* □ One off study * □

Other _______________________________________________

(b) within your MMA currently? ________________% Don’t know □

How have you estimated this? Expert judgement □ On-going scientific monitoring* □ One off study * □

Other ________________________________________________

8) What is the primary aim of the MMA? Please choose ONE.

Habitat conservation □ Cultural heritage □ Local economic development □ Species conservation □

Fisheries enhancement □ Education and/or research □ Recreation and tourism □ Other _____________________________

9) Is there an MMA management plan? Yes □ No □ In preparation □

10) Have there been any of the following schemes aimed at local fishers and other users, either run by your MMA, government departments or NGO or other international organisations, since the MMA was set up?

In the past Current

Small business grants or credit programs □ □

Alternative livelihood schemes □ □

Fisher micro-credit schemes or loans □ □

Cash compensation payments □ □

Boat or equipment buy-back schemes □ □

Benefit sharing projects □ □

Conflict resolution mechanisms □ □

MMA-associated development initiatives □ □

11) Does current MMA management include or involve any of the following (please tick all those that apply)?

Yes past Yes current Planned No Not sure

MMA formal staff training

(e.g. enforcement, monitoring, conflict resolution) □ □ □ □ □

Fisher compensation scheme(s) □ □ □ □ □

Fisheries management extending outside the MMA □ □ □ □ □

MMA designated as part of MMA network □ □ □ □ □

Collaborative monitoring/management with other MMAs □ □ □ □ □

National integrated coastal management plan(s) □ □ □ □ □

International monitoring or research initiative(s) □ □ □ □ □

International conservation grants or initiative(s) □ □ □ □ □

Education outreach initiative(s) □ □ □ □ □

Socio-economic monitoring □ □ □ □ □

Ecological monitoring □ □ □ □ □

Management effectiveness monitoring □ □ □ □ □

Wider endangered species protection initiative(s) □ □ □ □ □

Local MMA related community institution(s) □ □ □ □ □

National or International NGO(s) □ □ □ □ □

GEF project funding or technical assistance □ □ □ □ □

**Budgetary Information.**

12) What was the total value of all initial investments and grants made when the MMA was first set up?

Year __________ Currency______ Amount ____________ Don’t know □ None □

13) What was the total budget (from all sources) spent on MMA (including staff, management and tourism infrastructure, educational outreach etc) in 2005? Currency______ Amount __________ Don’t know □ None □

14) What proportion of management costs were covered by each of these source in 2005 (please make sure these add up to 100%)?

Government funds __ %

MMA generated revenues __ %

Donations and gifts __ %

National foundations or NGOs __ %

International foundations or NGOs __ %

15) Considering all the tools described above, what percentage of revenue generated goes to each of these uses:

Kept by MMA staff for MMA management costs __ %

Collected and kept by local community members / businesses __ %

Returned to central government office(s) or department(s) __ %

Other (please name) ______________________________ __ %

**MMA Uses and Benefits.**

16) Have you seen evidence in local fisheries of any of these things, since your MMA was set up? Please tick ALL those that apply. If you are not sure, or it is not applicable, please leave the boxes blank. *Fishing effort is staff and equipment investments and/or time spent by fishers. Harvest variance is the number or types of fish. Crowding refers to fishers being crowded close together*.

Inside MMA Near 5km the MMA Away from (>5km) the MMA

Less fishers □ □ □

More fishers □ □ □

Increased fishing effort □ □ □

Decreased fishing effort □ □ □

Increased catches □ □ □

Decreased catches □ □ □

Increased crowding □ □ □

Decreased crowding □ □ □

Increased harvest variance □ □ □

Decreased harvest variance □ □ □

17) Have you seen evidence of any of these things related to tourism within the MMA? Please tick all that apply. If you are not sure, or it is not applicable, please leave the boxes blank.

Increased tourism □

Increased stakeholder conflict □

Decreased stakeholder conflict □

Damage to coral from these visitors / users □

Erosion of local culture from MMA associated tourism □

Greater income / wealth for local communities □

MMA tourism-related pollution □

Greater local community employment opportunities □

Higher local prices for goods □

Great availability of goods □

Other (please describe) __________________________ □

18) Do fishers congregate on the edges of areas where fishing is not allowed?

Rarely/never □ Some fishers □ Most fishers □ Not applicable □

19) How many people work in each of the following types of employment in the area within 5km of MMA? Of these, how many are originally from the area (are local, meaning, have lived in the nearby area for more than 10 years)?

Number employed Of which are local

MMA paid staff, monitoring and research ___ ___

MMA tourism related employment ___ ___

Fishing inside or within 5km of the MMA ___ ___

20) How many of the following businesses are linked to the MMA? Please count total number of businesses and then fill in how many people are employed permanently in these businesses. Please mark this box if these are very rough estimates .

Number of businesses Number of people employed

Hotels / guest houses / resorts ___ ___

Dive shops / boat operators ___ ___

Fishing or diving guides ___ ___

Restaurants ___ ___

Tourist gift shops ___ ___

Tourist services ___ ___

Other ______________________ ___ ___

21) Which activities are allowed and prohibited within your MMA? *Limited activities include those only allowed in certain zones, or requiring permits and other restriction.*

Allowed Limited Not allowed No rules

Extractive uses:

Local commercial fishing □ □ □ □

Local subsistence fishing □ □ □ □

Foreign commercial fishing □ □ □ □

Blast fishing □ □ □ □

Cyanide fishing □ □ □ □

Coral mining □ □ □ □

Shell /ornamental species collection □ □ □ □

Sports fishing □ □ □ □

Mangrove wood collection □ □ □ □

Aquaculture □ □ □ □

Traditional hunting of protected species □ □ □ □

Extraction for building materials / medicines □ □ □ □

Other _______________________________□ □ □ □

Non extractive uses:

Boat anchoring / mooring □ □ □ □

Diving / snorkelling □ □ □ □

Photography □ □ □ □

Research/ monitoring □ □ □ □

Recreation □ □ □ □

Tourist tours, trips or visits □ □ □ □

Education □ □ □ □

Other ___________________________ □ □ □ □

22) Now, focusing on what takes place within the MMA, please look at this list of potential activities that occur. Please include both legal and illegal activities. This is the same list.

Extractive uses: Frequently Occasionally Never Don’t know

Local commercial fishing □ □ □ □

Local subsistence fishing □ □ □ □

Foreign commercial fishing □ □ □ □

Blast fishing □ □ □ □

Cyanide fishing □ □ □ □

Coral mining □ □ □ □

Shell /ornamental species collection □ □ □ □

Sports fishing □ □ □ □

Mangrove wood collection □ □ □ □

Aquaculture □ □ □ □

Traditional hunting of protected species □ □ □ □

Extraction for building materials / medicines □ □ □ □

Other _______________________________□ □ □ □

Non extractive uses:

Boat anchoring / mooring □ □ □ □

Diving / snorkelling □ □ □ □

Photography □ □ □ □

Research/ monitoring □ □ □ □

Recreation □ □ □ □

Tourist tours, trips or visits □ □ □ □

Education □ □ □ □

Other ___________________________ □ □ □ □

23) How many of each of these groups and how many in total visited your MMA, in 2005? *Please avoid double counting: fishers who also visit the MMA should only be counted in the fisher category.*

Local fishers (extract goods) ___

Local or national visitors (no extraction) ___

International visitors ___

Other (please specify)_________________________ ___ TOTAL: ___________________

Have these figures been estimated?

Based on entry fees/ permits □ Based on manger estimates □ Other ______________________ □

24) What are the main uses of the MMA by nearby communities? Please rank these in order, from 1 (most frequent use) to 5/6 (least frequent)? Frequency refers to number of visits occurring. If the use does not take place in the MMA, please leave mark the box 0.

Subsistence fishing ___

Commercial fishing ___

Collection of natural resources for food, building materials, medicines ___

Recreation ___

Cultural ceremonies or other cultural practises ___

Other ___________________________________ ___

**MMA Threats.**

25) Have you had any of the following in the last five years to a degree which has impacted the habitat quality within or nearby your MMA (please mark all that apply)?

Within the MMA Within 5km of MMA

Coral damage due to cyclones, hurricanes □ □

Sedimentation from on-land practises □ □

Chemical or agricultural pollution □ □

Large scale coastal development □ □

Coral bleaching □ □

Oil spill(s) □ □

Large scale local immigration □ □

War or civil unrest □ □

Natural disaster(s) □ □

Other (please specify) __________________________ □ □

26) Since the MMA was set up, how have these activities changed INSIDE the MMA:

Increased Decreased Stayed the same Don’t know None when established

Unsustainable/destructive fishing □ □ □ □ □

Large scale aquaculture □ □ □ □ □

Mangrove clearance □ □ □ □ □

Seabed drilling or mining □ □ □ □ □

Oil exploration □ □ □ □ □

Dredging, diking or filling □ □ □ □ □

Trawling □ □ □ □ □

Blast fishing □ □ □ □ □

Cyanide fishing □ □ □ □ □

Coral mining □ □ □ □ □

Catching endangered species □ □ □ □ □

Other __________________ □ □ □ □ □

Since the MMA was set up, have these activities OUTSIDE the MMA (this is the same list):

Increased Decreased Stayed the same Don’t know None when established

Unsustainable/destructive fishing□ □ □ □ □

Large scale aquaculture □ □ □ □ □

Mangrove clearance □ □ □ □ □

Seabed drilling or mining □ □ □ □ □

Oil exploration □ □ □ □ □

Dredging, diking or filling □ □ □ □ □

Trawling □ □ □ □ □

Blast fishing □ □ □ □ □

Cyanide fishing □ □ □ □ □

Coral mining □ □ □ □ □

Catching endangered species □ □ □ □ □

Other __________________ □ □ □ □ □

27) What do you consider to be the main threat to the MMA? Please note, this may be a threat not listed above.

Please describe _______________________________________________________________________________________________________

What, if any actions (if any) are being currently taken to address this threat? Please describe:_____________________________________________________________________________________________________________

_____________________________________________________________________________________________________________________

**MMA manager feedback.**

28) Have these ecological and socio-economic aspects changed within your MMA, in your opinion as result of the existence of the MMA?

Improved Worsened No change Not sure

(a) Habitat quality □ □ □ □

(b) Fisheries □ □ □ □

(c) Species conservation □ □ □ □

(d) Local economic development □ □ □ □

(e) Education and research □ □ □ □

(f) Cultural heritage □ □ □ □

(g) Other ___________________ □ □ □ □

29) What percentage of the MMA rule infractions (illegal activities) would you estimate are:

(a) Detected by MMA staff _________% (b) Punished _________ %

30) To what extent do you think the primary aim of the MMA, has been achieved, since the MMA was set up?

Not at all □ To limited extent □ To large extent □ Fully □

Do you think the MMA is currently a success in general? No □ Partially □ Mainly □ Very □

Do you have any additional comments you would like to add?___________________________________________________________________________

_____________________________________________________________________________________________________________________________

Please add your email address, if you would like a copy of the report: _____________________________________________________________________

If you are happy to share basic information in this questionnaire with Reefbase, please sign here: ______________________________________________

If you would like to contact me, my email is: [vhargreavesallen@gmail.com](mailto:vhargreavesallen@gmail.com).

**Thank you for your time**.
