# Supplementary material for: Drivers of coral reef marine protected area performance
Source: PLoS One. 2017 Jun 23;12(6):e0179394. doi: 10.1371/journal.pone.0179394 (PMC5482435; doi:10.1371/journal.pone.0179394)
Supplement: S1 Table — (DOCX) [file pone.0179394.s002.docx]

**S1 Table. Coding of the performance measures**

| **Outcome** | **Measures gleaned from questionnaire** | **Coding** |
| --- | --- | --- |
| Ecological | Change in live coral cover since established | Current cover – initial cover |
|  | Live coral cover compared to country average | Current cover- country average |
|  | Perceived changes in fisheries | - 1 = worse, 0 = no change, 1 = improvement |
|  | Perceived changes in species conservation | - 1 = worse, 0 = no change, 1 = improvement |
| Social | Perceived change in stakeholder conflict | - 1 = worse, 0 = no change, 1 = improvement |
| Economic | Perceived greater wealth for local communities as a result of MPA | 0 = no change, 1 = improvement |
|  | Estimated Number jobs supported per km^2^ | Total of jobs supported by each industry |
| Threats | Number of destructive activities that have decreased inside the MPA over time | Number when established – current number |
|  | Difference between number of large scale threats inside and outside MPA | Number outside – number inside |
|  | Number of destructive activities to stay the same / decreased inside, but not outside MPA | Number decreased inside – number decreased outside |
| Goals | Number of banned activities occurring | No. banned activities – number actually occurring (occasionally or frequently) |
|  | Perceived extent of primary aim achieved | Ordinal scale, 1 to 4 |
|  | Perceived success of the MPA in general | Ordinal scale, 1 to 4 |
