# Supplementary material for: Drivers of coral reef marine protected area performance
Source: PLoS One. 2017 Jun 23;12(6):e0179394. doi: 10.1371/journal.pone.0179394 (PMC5482435; doi:10.1371/journal.pone.0179394)
Supplement: S2 Table — (DOCX) [file pone.0179394.s003.docx]

**S2 Table. Source and units for explanatory variables used in regressions**

|  | **Variable** | **Source** | **Unit** |
| --- | --- | --- | --- |
| MPA features | - Size | survey response | km^2^ |
|  | No take area | survey response | dummy |
|  | Size no-take area | survey response | km^2^ |
|  | Age | survey response | years since designation |
|  | - No. zones | survey response | number |
|  | World Heritage Site | survey response | dummy |
|  | Management: Government, community, NGO, multiple | survey response | dummy |
|  | Low / high IUCN number | survey response | dummy |
|  | Mooring buoys | survey response | dummy |
| Aims | Primary aim of MPA: habitat protection, fisheries, tourism or multiple | survey response | dummy |
| Management actions | - Number of staff | survey response | number |
|  | Staff per km^2^ | no. staff / area | no. / km^2^ |
|  | Past / current development related tools: business grants, alternative livelihood schemes, micro-credit, compensation, fishing buy-back schemes, benefit sharing projects, conflict resolution measures, development projects | survey response | dummy |
|  | Past or current management activities e.g. fisheries management, international monitoring program, education/ outreach, etc | survey response | dummy |
|  | Management plan | survey response | dummy |
|  | No. regulated activities | total from survey list | number |
|  | No. banned activities | total from survey list | number |
|  | % illegal activities detected | survey response | % |
|  | % detected punished | survey response | % |
|  | % illegal activities punished | % detected * % punished | % |
| Financial | - Value initial investment | survey response | US$2005 |
|  | Current budget | survey response | US$2005 |
|  | Budget per km^2^ | budget / area | US$2005 / km^2^ |
|  | % budget from government, revenues, donations, national & international NGOs | survey response | % |
|  | % funds used for management costs, local projects, returned to government | survey response | % |
| Threats / uses | - Number fishers | survey response | number |
|  | - Fishing pressure | no. reported / area | number / km^2^ |
|  | - No. large threats inside / outside | survey response | number |
|  | - No. visitors | survey response | number |
|  | - Visitor pressure | no. visitors / area | number / km^2^ |
|  | - Rank of local uses (subsistence fishing, commercial fishing, recreational) | survey response | rank |
|  | - Main threat originates inside MPA / outside MPA | main threat type | dummy |
|  | - Coral damage from tourists | survey response | dummy |
|  | - Suitability of action to main threat | main threat type | rank |
| Local context | Increased tourism due to MPA | survey response | dummy |
|  | Percentage jobs to locals | survey response | % |
|  | Percentage management jobs to locals | survey response | % |
|  | - Increased economic development due to MPA | survey response | dummy |
|  | - Erosion of local culture | survey response | dummy |
| National context | Gross domestic product per capita (GDP pc) | CIA factbook | US$ |
|  | National population density | CIA factbook | number / km^2^ |
|  | Human development index 2005 | UNDP | HDI value (0-1) |
|  | Human Poverty Index 2005 | UNDP | number |
|  | Annual marine production | Earthtrends | Millions tons |
|  | Number of marine protected areas | WCPA | number |
|  | Area within MPAs | WCPA | number / km^2^ |
|  | Fish protein as percentage of diet | Earthtrends | % |
|  | National % of reefs at high risk in 1998 | Burke and others, 1998 | % |
|  | National % at high and threatened risk in 1998 | Burke and others, 1998 | % |
| Region | Pacific, Asia, Africa, Americas | location | dummy |
|  | Developing country | location | dummy |
| Survey variables | - Respondent job type (management, government body, NGO or other) | survey response | dummy |
|  | - Coral data source type for past and current coral cover (monitoring, study or manager estimate) | survey response | dummy |
|  | - Coral cover data supplemented | if applicable | dummy |
