# Supplementary material for: Drivers of coral reef marine protected area performance
Source: PLoS One. 2017 Jun 23;12(6):e0179394. doi: 10.1371/journal.pone.0179394 (PMC5482435; doi:10.1371/journal.pone.0179394)
Supplement: S3 Table — (DOCX) [file pone.0179394.s004.docx]

**S3 Table. Sample population statistics (N=66 unless otherwise stated)**

| **Variable** | **Mean** | **Median** | **St Dev** | **Range** |
| --- | --- | --- | --- | --- |
| **MPA Age (years)** | 14.4 | 12 | 10.7 | 1 to 69 |
| **Size (km^-2^)** | 9,713 | 75.3 | 45,840 | 0.09 to 344,000 |
| **No take area (km^-2^)** | 2,260 | 0.2 | 14,696 | 0 to 115,395 |
| **No. zones** | 2.1 | 2 | 1.9 | 0 to 5+ |
| **Set-up budget (US$’000)** | 266.6 | 13.1 | 549 | 0 to 2,546 |
| **Overall budget (US$’000) N= 40** | 648.4 | 97.1 | 1,809 | 0 to 12,000 |
| **Budget km^-2^ (US$’000) N= 56** | 240.1 | 1.8 | 1,519 | 0 to 11,300 |
| **Management budget km^-2^ (US$’000)** | 229.5 | 1.1 | 1.6 | 0 to 11,300 |
| **Visitor pressure (km^-2^)** | 74,666 | 77.4 | 39,405 | 0.003 to 2,750,000 |
| **Fisher pressure (km^-2^)** | 224 | 1.4 | 829 | 0 to 4,688 |
