# Supplementary material for: Drivers of coral reef marine protected area performance
Source: PLoS One. 2017 Jun 23;12(6):e0179394. doi: 10.1371/journal.pone.0179394 (PMC5482435; doi:10.1371/journal.pone.0179394)
Supplement: S4 Table — Standardised coefficients are given, together with model summaries. For explanation of the explanatory variables see S4 Table. * = p<0.1, ** = p<0.05, *** = p<0.001. (DOCX) [file pone.0179394.s005.docx]

**S4 Table. Results of ordered logit regressions to determine significant variables related to achievement of MPAs aims and overall success.** Standardised coefficients are given, together with model summaries. For explanation of the explanatory variables see Table S4. *=p<0.1, **=p<0.05, ***=p<0.001.

|  | **Achievement of Primary Aim** | | **Perceived MPA success** | |
| --- | --- | --- | --- | --- |
| MPA features | No. zones | 1.286 *** | No. zones | 3.405 ** |
|  | Age (years) | -0.115 | No take area | -7.856 ** |
|  | Size (km2) | 0.0002 ** |  |  |
|  | Age * size | -0.0001** |  |  |
| Aims | Tourism primary aim | 3.15 ** |  |  |
|  | Multiple aims | -3.89 *** |  |  |
| Management actions | Staff per km^2^ | 0.14*** | % illegal activities punished | 0.2098 ** |
|  | Benefit sharing project(s) | 5.72*** | Affiliated community institution(s) | 7.498 ** |
|  | Development initiative(s) | 4.31*** |  |  |
|  | No. banned activities | 0.99*** |  |  |
| Financial |  |  | % funding returned to government | -0.119 ** |
|  |  |  | % funding from donations | 0.264 ** |
|  |  |  | Funding per km^2^ | 0.001* |
| Threats / uses | No. threats inside MPA | -0.712 ** |  |  |
| National context | GDP pc ppp | 0.0002 *** | GDP pc ppp | 0.0002* |
|  | % reefs at high risk | -0.068 *** | % reefs at high risk | -0.165** |
| Region |  |  | In Asia | 14.131 ** |
|  | N  LR chi^2^  Prob > chi^2^  Adj R^2^ | 60  97.74  0.000  0.671 | N  LR chi^2^  Prob > chi^2^  Adj R^2^ | 46  91.53  0.000  0.773 |
