# Supplementary material for: Drivers of coral reef marine protected area performance
Source: PLoS One. 2017 Jun 23;12(6):e0179394. doi: 10.1371/journal.pone.0179394 (PMC5482435; doi:10.1371/journal.pone.0179394)
Supplement: S5 Table — * = p<0.1, ** = p<0.05, *** = p<0.001. (DOCX) [file pone.0179394.s006.docx]

**S5 Table. Regression to determine significant variables related to temporal changes in live coral cover (ordinary least squares) and improvements in fisheries (logistic).** *=p<0.1, **=p<0.05, ***=p<0.001.

|  | **Temporal change coral** | | **Improvement in fisheries** | |
| --- | --- | --- | --- | --- |
|  | Constant | -2.001 | Constant | -91.4* |
| **MPA features** | Age | -0.44*** | No-take area | -11.27** |
|  | No. staff | -0.12*** |  |  |
|  | No. zones | 1.77** |  |  |
|  | Co-management | -9.04*** |  |  |
| **Management actions** | Management plan | 13.41*** | Compensation | 16.81* |
|  | Fisher compensation | 5.85*** | % illegal activities detected | 0.27* |
|  |  |  | Community institutions | 18.6* |
|  |  |  | No regulated activities | 1.217* |
| **Financial** | % funds used for management costs | 0.10** |  |  |
| **Threats / Use** | No. threats inside | -3.84*** | No. threats inside | -1.75** |
|  | Rank subsistence fishing | -2.62** |  |  |
| **Local context** | Coastal zone management | 5.85** | Increased tourism | 19.48* |
| **National context** |  |  | Human development index | 98.98* |
| **Region** | Asia | 15.44*** | Americas | -15.47* |
| **Model Parameters** | N  F  Prob > F  Adj R^2^ | 40  8.47  0.000  0.678 | N  LR chi^2^  Prob > chi^2^  Adj R^2^ | 59  59.96  0.000  0.769 |
