# Supplementary material for: Drivers of coral reef marine protected area performance
Source: PLoS One. 2017 Jun 23;12(6):e0179394. doi: 10.1371/journal.pone.0179394 (PMC5482435; doi:10.1371/journal.pone.0179394)
Supplement: S6 Table — * = p<0.1, ** = p<0.05, *** = p<0.001. (DOCX) [file pone.0179394.s007.docx]

**S6 Table. Logit regressions to determine significant variables related to increase in wealth and decrease in conflict at MPAs.** *=p<0.1, **=p<0.05, ***=p<0.001.

|  | **Increase in wealth** | | **Conflict has decreased** | |
| --- | --- | --- | --- | --- |
|  | Constant | -9.78 | Constant | -8.78** |
| **MPA features** | No-take area | -11.98* | Size no-take | 0.0002** |
|  | High IUCN category | -16.98* | Community managed | 3.99* |
| **Primary Aim** | Multiple aims | -38.46* |  |  |
| **Management actions** | Fisheries management | 15.01* | Alternative livelihood project | 7.31* |
|  | No. activities banned | -4.73* |  |  |
|  | Management plan | 39.39* |  |  |
|  | % illegal activities detected | 0.26** |  |  |
| **Financial** |  |  | % funds from intl. organizations | 0.084** |
| **Threats / Use** |  |  | No. threats inside | -3.06** |
| **National context** | LDC | 17.69** | % reefs at high risk | 0.090** |
| **Region** | Pacific | 46.56* |  |  |
| **Model Parameters** | N  LR chi^2^  Prob > chi^2^  Adj R^2^ | 57  64.4  0.0000  0.826 | N  LR chi^2^  Prob > chi^2^  Adj R^2^ | 48  47.8  0.0000  0.745 |
