# Supplementary material for: Drivers of coral reef marine protected area performance
Source: PLoS One. 2017 Jun 23;12(6):e0179394. doi: 10.1371/journal.pone.0179394 (PMC5482435; doi:10.1371/journal.pone.0179394)
Supplement: S7 Table — * = p<0.1, ** = p<0.05, *** = p<0.001. (DOCX) [file pone.0179394.s008.docx]

**S7 Table. Ordinary Least Squares regressions to determine significant variables related to the natural log of the number of destructive activities decreasing over time and the number of large scale threats inside the MPA compared to outside.** *=p<0.1, **=p<0.05, ***=p<0.001.

|  | **No. destruct. activities decreasing** | | **No threats compared to outside** | |
| --- | --- | --- | --- | --- |
|  | Constant | -0.67*** | Constant | -1.86*** |
| **MPA features** | Age | 0.009*** | Age^2^ | -0.00004*** |
|  | Size | 0.0002*** | Size no-take | 0.0004*** |
|  | Age * size | -0.0002*** |  |  |
|  | Size no-take | 0.002*** |  |  |
|  | Mooring buoys | 0.297*** |  |  |
| **Management actions** | Freq. research / monitoring | -0.163*** | % illegal activities punished | 0.013** |
|  |  |  | Staff per km^2^ | 0.050*** |
|  |  |  | Fisheries management | 1.29*** |
| **Financial** | Intl conservation grant | 0.195*** | (Funding per km^2^)^2^ | -0.000001*** |
|  |  |  | % funds used for management costs | 0.010*** |
| **Threats/Use** | Rank commercial fishing | 0.04** | Rank commercial fishing | -0.145** |
| **Local context** |  |  | Coastal zone management | -0.59** |
| **National context** | % Reefs at risk | 0.007*** |  |  |
| **Region** | Asia | 0.19*** |  |  |
| **Survey variables** |  |  | NGO employee | 0.795*** |
| **Model Parameters** | N  F  Prob > F  Adj R^2^ | 49  10.7  0.0000  0.668 | N  F  Prob > F  Adj R^2^ | 39  14.6  0.000  0.839 |
