# Supplementary material for: Drivers of coral reef marine protected area performance
Source: PLoS One. 2017 Jun 23;12(6):e0179394. doi: 10.1371/journal.pone.0179394 (PMC5482435; doi:10.1371/journal.pone.0179394)
Supplement: S8 Table — This table summarises the variables used in this study that been shown quantitatively or anecdotally to influence MPA performance in the literature. For expected direction of the impact on performance, NL denotes a non-linear relationship, + a positive and–a negative. References are listed below. (DOCX) [file pone.0179394.s009.docx]

**S8 Table. Variables hypothesized to impact facets of MPA success**. This table summarises the variables used in this study that been shown quantitatively or anecdotally to influence MPA performance in the literature. For expected direction of the impact on performance, NL denotes a non-linear relationship, + a positive and – a negative. References are listed below.

|  | **MPA / management variable** | **Expected direction of relationship** | **Study** |
| --- | --- | --- | --- |
| MPA features | MPA size | NL / + | [1-12] |
|  | Existence or size of no-take area | + | [2, 4, 9, 13-18] |
|  | Age | NL / + | [4, 9, 10, 19] |
|  | Low IUCN number | + | [20-22] |
|  | Zoning | + | [23-25] |
|  | Community managed | + / - | [26-28] |
|  | Government managed | - | [26, 29, 30] |
|  | Multiple / co-management | + | [23, 29, 31-35] |
|  | Part of physical or monitoring MPA network | + | [3, 6, 36-43] |
| Aims | Multiple aims | - | [23, 26, 44, 45] |
| Management actions | Existence management plan | + | [24, 46] |
|  | No. staff / level activity | + | [19, 47, 48] |
|  | Staff training | + | [36, 49, 50] |
|  | No. regulations or bans on destructive activities | + | [26, 46, 48] |
|  | % activities detected and/or enforced | + | [9, 26, 46, 50-59] |
|  | Community involvement and/or consultation | + | [19, 24, 26, 60-65] |
|  | Resource user participation, community institutions | + | [18, 31, 52, 55, 61, 66-69] |
|  | Incentive based programs / alternative livelihoods | + | [36, 59, 61, 70-72] |
|  | Environmental education and outreach | + | [23, 24, 29, 31, 54, 56, 73, 74] |
|  | Conflict resolution mechanisms | + | [23, 26, 44, 46, 52, 75] |
|  | Social and ecological monitoring | + | [12, 19, 24, 37, 49, 55, 72, 75-78] |
|  | Management effectiveness evaluation /adaptive management | + | [11, 16, 19, 24, 79-82] |
|  | Technical supervision / advice from outside organization e.g. NGO | + | [18, 23, 37, 61] |
|  | Compensation to groups suffering user costs | + | [34, 47, 61, 83, 84] |
| Financial | MPA funding (absolute / per area / for active management) | + | [18, 48, 49, 85-90] |
|  | Extent facilities, equipment and infrastructure | + | [19, 55, 72, 88, 90, 91] |
|  | % funding from user fees | + / - | [23, 87, 92, 93] |
|  | Funding to local community projects | + | [66, 72, 89, 92, 94] |
| Threats / uses | No. threats inside or outside | - | [13, 22, 37, 95-98] |
|  | Number of fishers / fishing pressure | - | [3, 11, 21, 46, 99, 100] |
|  | Number of visitors/ visitor pressure | + / - | [3, 8, 101-103] |
| Local / national context | Equitable distribution of benefits locally | + | [19, 37, 44, 52, 60, 66, 104, 105] |
|  | Increased tourism | + / - | [101, 102, 106-112] |
|  | High degree poverty or natural resource dependence | - | [54, 63, 75, 105, 113, 114] |
|  | Coastal zone management beyond MPA | + | [13, 18, 22, 23, 27, 39, 95, 115-118] |
|  | Fisheries management in addition to MPA | + | [24, 27, 36, 45, 119, 120] |
|  | GDP pc / economic development / more developed country (MDC) | + | [11, 31, 89, 121] |
|  | Human development index (HDI) | + / NL | [11, 19, 31, 88, 122] |
|  | % reefs at risk | + / - | [37, 123] |
| Region | Americas, Asia | - | [95, 123] |
|  | Pacific | + | [95, 123] |
| Survey variable | Respondent is part of management staff | + | [124] |

References for S8 Table.

1. Alder J, Zeller D, Pitcher T, Sumaila R. A method for evaluating marine protected area management. Coastal Management. 2002;30(2):121-31.

2. Lauck T, Clark CW, Mangel M, Munro GR. Implementing the precautionary principle in fishries management through marine reserves. Ecological Applications. 1998;8(1):S72-S8.

3. Lowry GK, White AT, Christie P. Scaling Up to Networks of Marine Protected Areas in the Philippines: Biophysical, Legal, Institutional, and Social Considerations. Coastal Management. 2009;37(3):274-90.

4. Claudet J, Osenberg CW, edetti-Cecchi L, Domenici P, Garcia-Charton JA, Perez-Ruzafa A, et al. Marine reserves: size and age do matter. Ecology Letters. 2008;11(5):481-9.

5. Mora C, Andrefouet S, Costello MJ, Kranenburg C, Rollo A, Veron J, et al. Coral reefs and the global network of marine protected areas. Science. 2006;312(5781):1750-1.

6. Halpern BS, Warner RR. Matching marine reserve design to reserve objectives. Proceedings of the Royal Society of London Series B-Biological Sciences. 2003;270:1871-8.

7. Weeks R, Russ G, Alcala AC, White AT. Effectiveness of Marine Protected Areas in the Philippines for Biodiversity Conservation. Conservation Biology. 2009;24(2):531-40.

8. Roberts CM, Hawkins JP. Fully-proteced marine reserves: a guide. Washington DC and York, UK: WWF and University of York, 2000.

9. Edgar GJ, Stuart-Smith RD, Willis TJ, Kininmonth S, Baker SC, Banks S, et al. Global conservation outcomes depend on marine protected areas with five key features. Nature. 2014;506:216-20.

10. Vandeperre F, Higgins RM, Sanchez-Meca J, Maynou F, Goni R, Martin-Sosa P, et al. Effects of no‐take area size and age of marine protected areas on fisheries yields: a meta‐analytical approach. Fish and Fisheries. 2011;12:412-26.

11. Fox HE, Holtzman JL, Haisfield KM, McNally CG, Cid GA, Mascia MB, et al. How Are Our MPAs Doing? Challenges in Assessing Global Patterns in Marine Protected Area Performance. Coastal Management. 2014;42(3):207-26.

12. Lester SE, Halpern BS, Grorud-Colvert K, Lubchenco J, Ruttenberg BI, Gaines SD, et al. Biological effects within no-take marine reserves: a global synthesis. Marine Ecology Progress Series. 2009;384:33-46.

13. Boersma PD, Parrish JK. Limiting abuse: marine protected areas, a limited solution. Ecological Economics. 1999;31(2):287-304.

14. Sumaila UR. Protected marine reserves as fisheries management tools: a bioeconomic analysis. Fisheries Research. 1998;37(1-3):287-96.

15. Hughes TP, Bellwood DR, Folke CS, McCook LJ, Pandolfi JM. No-take areas, herbivory and coral reef resilience. Trends in Ecology & Evolution. 2007;22(1):1-3.

16. Agardy T, Bridgewater P, Crosby MP, Day J, Dayton PK, Kenchington R, et al. Dangerous targets? Unresolved issues and ideological clashes around marine protected areas. Aquatic Conservation: Marine and Freshwater Ecosystems. 2003;13(4):353-67.

17. Noel J, Weigel J. Marine protected areas: from conservation to sustainable development. International Journal of Sustainable Society. 2007;10(3).

18. White AT, Courtney CA, Salamanca A. Experience with marine protected area planning and management in the Philippines. Coastal Management. 2002;30(1):1-26.

19. Leverington F, Hockings M, Lemos Costa K. Management effectiveness evaluation in protected areas: Report for the project: Global study into management effectiveness evaluation of protected areas. Queensland, Australia: The University of Gatton, IUCN WCPA, TNC, WWF; 2008.

20. Naughton-Treves L, Holland MB, Brandon K. The role of protected areas in conserving biodiversity and sustaining local livelihoods. Annual Review of Environment and Resources. 2005;30:219-52.

21. Pollnac R, Christie P, Cinner JE, T. D, Daw TM, Forrester GE, et al. Marine reserves as linked social-ecological systems. Proceedings of the National Academy of Sciences of the United States. 2010;107(43):18262–5.

22. Guarderas AP, Hacker SD, Lubchenco J. Current Status of Marine Protected Areas in Latin America and the Caribbean. Conservation Biology. 2008;22(6):1630-40.

23. Christie P, White AT. Best practices for improved governance of coral reef marine protected areas. Coral Reefs. 2007;26(4):1047-56.

24. Tupper M, Oliver J, Kenchington R, McClanahan T, Muthiga N, Gill D, et al. Lessons learned and best practices in the management of coral reefs. Penang, Malaysia: World Fish Centre., 2008.

25. Ruiz-Frau A, Kaiser MJ, Edwards-Jones G, Klein CJ, Segan DB, Possingham HP. Balancing extractive and non-extractive uses in marine conservation plans. Marine Policy. 2015;52:11-8.

26. Pomeroy R, Mascia M, Pollnac R. Marine protected areas: the social dimension. FAO fisheries report. 2007;(No.825):149-81.

27. Christie P, White A, Deguit E. Starting point or solution? Community-based marine protected areas in the Philippines. Journal of Environmental Management. 2002;66(4):441-54.

28. Alcala AC, Russ GR. No-take Marine Reserves and Reef Fisheries Management in the Philippines: A New People Power Revolution. AMBIO: A Journal of the Human Environment. 2006;35(5):245-54.

29. Clifton J. Prospects for co-management in Indonesia’s marine protected areas. Marine Policy. 2003;27:389-95.

30. Hilborn R, Orensanz JM, Parma AM. Institutions, incentives and the future of fisheries. Philosophical Transactions of the Royal Society B-Biological Sciences. 2005;360(1453):47-57.

31. Cinner J, McClanahan TR, Daw TM, Graham NAJ, Maina J, Wilson SK, et al. Linking Social and Ecological Systems to Sustain Coral Reef Fisheries. Current Biology. 2009;19(3):206-12.

32. Jones PJS. Marine protected area strategies: issues, divergences and the search for middle ground. Reviews in Fish Biology and Fisheries. 2001;11(3):197-216.

33. Oracion EG, Miller ML, Christie P. Marine protected areas for whom? Fisheries, tourism, and solidarity in a Philippine community. Ocean & Coastal Management. 2005;48(3-6):393-410.

34. Berkes F. From Community-Based Resource Management to Complex Systems: The Scale Issue and Marine Commons. Ecology and Society. 2006;11(1).

35. Gutierrez NL, Hilborn R, Defeo O. Leadership, social capital and incentives promote successful fisheries. Nature. 2011;470:386-9.

36. Wilkinson C. Status of coral reefs of the world: 2004. Townsville, Queensland, Australia.: Australian Institute of Marine Science (AIMS). 2004.

37. Jameson SC, Tupper MH, Ridley JM. The three screen doors: can marine "protected" areas be effective? Marine Pollution Bulletin. 2002;44(11):1177-83.

38. World B. Scaling up Marine Management: The Role of Marine Protected Areas. Washington, DC USA: Environment Department/Sustainable Development Network, 2006.

39. Iucn W. Establishing Networks of Marine Protected Areas: A Guide for Developing National and Regional Capacity for Building MPA Networks. IUCN, 2007.

40. Palumbi SR. Marine reserves and ocean neighborhoods: The spatial scale of marine populations and their management. Annual Review of Environment and Resources. 2004;29:31-68.

41. Roberts CM, Branch G, Bustamante RH, Castilla JC, Dugan J, Halpern BS, et al. Application of ecological criteria in selecting marine reserves and developing reserve networks. Ecological Applications. 2003;13(1):S215-S28.

42. Ferse SCA, Costa MM, Manez KS, Adhuri DS, Glaser M. Allies, not aliens: increasing the role of local communities in marine protected area implementation. Environmental Conservation. 2010;37(1):23-34.

43. Horigue V, Aliño PM, White AT, Pressey RL. Marine protected area networks in the Philippines: trends and challenges for establishment and governance. Ocean and Coastal Management. 2012;64:15-26.

44. Christie P, editor Marine protected areas as biological successes and social failures in southeast Asia. Aquatic Protected Areas as Fisheries Management Tools; 2004: American fisheries society symposium series.

45. Gaines SD, White C, Carr MH, Palumbi SR. Designing marine reserve networks for both conservation and fisheries management. Proceedings of the National Academy of Sciences of the United States. 2010;107(43):18286-93.

46. Halls AS, Burn RW, Abeyasekera A. Interdisciplinary Mulivariate Analysis for Adaptive Co-Management. London: Department for International Development, 2002.

47. Bruner A, Gullison RE, Rice R, da Fonseca G. Effectiveness of Parks in Protecting Tropical Biodiversity. Science. 2001;291(5501):125-8.

48. Gill DA, Mascia MB, Ahmadia GN, Glew L, Lester SE, Barnes M, et al. Capacity shortfalls hinder the performance of marine protected areas globally. Nature. 2017;543(7647):665-9.

49. Kelleher G. A global representative system of marine protected areas. Ocean & Coastal Management. 1996;32(2):123-6.

50. Kelleher G, Recchia C. Lessons from marine protected areas around the world. Parks. 1998;8(2):1-4.

51. Ehler CN. Indicators to measure governance performance in integrated coastal management. Ocean and Coastal Management. 2003;46(3-4):335-45.

52. Ostrom E. Governing the Commons: The Evolution of Collective Action: Cambridge University Press.; 1990.

53. Walmsley SF, White AT. Influence of social, management and enforcement factors on the long-term ecological effects of marine sanctuaries. Environmental Conservation. 2003;30(4):338-407.

54. McClanahan T, Davies J, Maina J. Factors influencing resource users and managers' perceptions towards marine protected area management in Kenya. Environmental Conservation. 2005;32(1):42-9.

55. Lundquist CJ, Granek EF. Strategies for successful marine conservation: Integrating socioeconomic, political, and scientific factors. Conservation Biology. 2005;19(6):1771-8.

56. Stoll-Kleemann S, Bender S, Berghîfer A, Bertzky M, Fritz-Vietta N, Schliep R, et al. Linking Governance and Management Perspectives with Conservation Success in Protected Areas and Biosphere Reserves. Berlin, Germany: Humboldt Unoversity, 2006.

57. Mascia MB, Claus CA, Naidoo R. Impacts of marine protected areas on fishing communities. Conservation Biology. 2010;24:1424-9.

58. Bergseth BJ, Russ GR, Cinner JE. Measuring and monitoring compliance in no-take marine reserves. Fish and Fisheries. 2015;16(2):240-58.

59. Kaplan KA, Ahmadia GN, Fox HE, Glew L, Pomeranz EF, Sullivan P. Linking ecological condition to enforcement of marine protected area regulations in the greater Caribbean region. Marine Policy. 2015;62:186-95.

60. Mascia M. Social Dimensions of Marine Reserves. In: Dahlgren C, Sobel J, editors. Marine Reserves: A Guide to Science, Design, and Use. Washington, DC.: Island Press; 2004. p. 164-86.

61. Pollnac RB, Crawford BR, Gorospe MLG. Discovering factors that influence the success of community-based marine protected areas in the Visayas, Philippines. Ocean & Coastal Management. 2001;44(11-12):683-710.

62. Christie P, Pollnac RB, Oracion EG, Abonsolin A, Diaz R, Pietri D. Back to Basics: An Empirical Study Demonstrating the Importance of Local-Level Dynamics for the Success of Tropical Marine Ecosystem-Based Management. Coastal Management. 2009;37(3):349-73.

63. McClanahan T, Marnane MJ, Cinner JE, Kiene WE. A Comparison of Marine Protected Areas and Alternative Approaches to Coral-Reef Management. Current Biology. 2006;16(14):1408-13.

64. Bunce L, Townsley P, Pomeroy RS, Pollnac R. Socioeconomic manual for coral reef management. GCNRM / IUCN / Australian Institute of Marine Science, 2000.

65. Dalton T, Forrester GE, Pollnac R. Participation, Process Quality, and Performance of Marine Protected Areas in the Wider Caribbean. Environmental Management. 2012;49(6):1224-37.

66. Charles A, Wilson L. Human dimensions of Marine Protected Areas. Ices Journal of Marine Science. 2009;66(1):6-15.

67. Pollnac R, Seara T. Factors influencing success of marine protected areas in the Visayas, Philippines as related to increasing protected area coverage. Environmental Management. 2011;47(4):584-92.

68. Jentoft S, van Son TC, Bjorkan M. Marine protected areas: A governance system analysis. Human Ecology. 2007;35:611-22.

69. Brown K, Adger WN, Tompkins E, Bacon P, Shim D, Young K. Trade-off analysis for marine protected area management. Ecological Economics. 2001;37(3):417-34.

70. Pomeroy RS, Oracion E, Pollnac R, Caballes C. Perceived economic factors influencing the sustainability of integrated coastal management projects in the Philippines. Ocean & Coastal Management. 2005;48:360-77.

71. Christie P. Observed and perceived environmental impacts of marine protected areas in two Southeast Asia sites. Ocean and Coastal Management. 2005;48(3-6):252-70.

72. Butardo-Toribio MZ, Aliño PM, Guiang ES. Cost-Benefit Study of Marine Protected Areas: Implications on Financing and Institutional Needs. The Philippine Agricultural Scientist. 2009;92(2).

73. Browning LJ, Finlay RAO, Fox LRE. Education as a tool for coral reef conservation: lessons from marine protected areas. In: Cote IM, Reynolds JD, editors. Coral Reef Conservation. Conservation Biology. Cambridge: Cambridge University Press; 2006. p. 419-54.

74. Leisher C, Mangubhai S, Hess S, Widodo H, Soekirman T, Tjoe S, et al. Measuring the benefits and costs of community education and outreach in marine protected areas. Marine Policy. 2012;36(5):1005-11.

75. Fox HE, Mascia MB, Basurto X, Costa A, Glew L, Heinemann D, et al. Reexamining the science of marine protected areas: linking knowledge to action. Conservation Letters. 2012;5(1):1-10.

76. Christie P. Creating space for interdisciplinary marine and coastal research: five dilemmas and suggested resolutions. Environmental Conservation. 2011;32(2):172-86.

77. Day J. The need and practice of monitoring, evaluating and adapting marine planning and management - lessons from the Great Barrier Reef. Marine Policy. 2008;32(5):823-31.

78. Ban NC, Cinner JE, Adams VM, Mills M, Almany GR, Ban SS, et al. Recasting shortfalls of marine protected areas as opportunities through adaptive management. Aquatic Conservation: Marine and Freshwater Ecosystems. 2012;22:262-71.

79. Pomeroy RS, Watson LM, Parks JE, Cid GA. How is your MPA doing? A methodology for evaluating the management effectiveness of marine protected areas. Ocean & Coastal Management. 2004;48(7-8):485-502.

80. Ervin J. Protected area assessments in perspective. Bioscience. 2003;53(9):819-22.

81. Warner TE, Pomeroy RS. Creating compliance: a cross-sectional study of the factors associated with Marine Protected Area outcomes. Marine Policy. 2012;36:922-32.

82. Holling CS. Understanding the Complexity of Economic, Ecological, and Social Systems. Ecosystems. 2001;4(5):390-405.

83. Rettig B. Who should preserve the marine environment? Marine Resource Economics. 1994;9:87-94.

84. Emerton L. Economic tools for the Management of Marine Protected Areas in Eastern Africa. IUCN, East Afric Office, Nairobi., 1999.

85. Gladstone W. The ecological and social basis for management of a Red Sea marine-protected area. Ocean & Coastal Management. 2000;43(12):1015-32.

86. White AT, Eisma-Osorio RL, Green SJ. Integrated coastal management and marine protected areas: Complementarity in the Philippines. Ocean & Coastal Management. 2005;48(11-12):948-71.

87. Gravestock P, Roberts CM, Bailey A. The income requirements of marine protected areas. Ocean & Coastal Management. 2008;51(3):272-83.

88. Bruner AG, Gullison RE, Balmford A. Financial costs and shortfalls of managing and expanding protected-area systems in developing countries. Bioscience. 2004;54(12):1119-26.

89. Balmford A, Gaston KJ, Blyth S, James A, Kapos V. Global variation in terrestrial conservation costs, conservation benefits, and unmet conservation needs. Proceedings of the National Academy of Sciences of the United States of America. 2003;100(3):1046-50.

90. Ervin J. Rapid assessment of protected area management effectiveness in four countries. Bioscience. 2003;53(9):833-41.

91. Rosero OR. An Analysis of the Law Enforcement Chain in the Eastern Tropical Pacific Seascape. San Francisco, USA: WildAid, Inc., Conservation International, 2010.

92. Emerton L, Bishop J, Thomas B. Sustainable Financing of Protected Areas. A global review of challenges and options. Gland, Switzerland: IUCN; 2006.

93. Depondt F, Green E. Diving user fees and the financial sustainability of marine protected areas: Opportunities and impediments. Ocean & Coastal Management. 2006;49(3-4):188-202.

94. Niesten E, Gjertsen H. Economic Incentives for Marine Conservation. Virginia, USA: Conservation International, Division SaK; 2010.

95. Wilkinson C. Status of the reefs of the word: summary of threats and remdial action. In: Cote IM, Reynolds JD, editors. Coral Reef Conservation. Conservation Biology.: Cambridge.; 2006. p. 3-39.

96. Allison GW, Lubchenco J, Carr MH. Marine reserves are necessary but not sufficient for marine conservation. Ecological Applications. 1998;8(1):S79-S92.

97. Keller BD, Gleason DF, McLeod E, Woodley CM, Airame S, Causey BD, et al. Climate Change, Coral Reef Ecosystems, and Management Options for Marine Protected Areas. Environmental Management. 2009;44(6):1069-88.

98. Bunce M, Brown K, Rosendo S. Policy misfits, climate change and cross-scale vulnerability in coastal Africa: how development projects undermine resilience. Environmental Science and Policy. 2010;13(6):485-97.

99. Roberts CM, Polunin NVC. Are marine reserves effective in management of reef fisheries? Reviews in Fish Biology and Fisheries. 1991;1(1):65-91.

100. Le Quesne WJF. Are flawed MPAs any good or just a new way of making old mistakes? ICES Journal of Marine Science: Journal du Conseil. 2009;66(1):132-6.

101. Dixon JA, Scura LF, Vanthof T. Meeting Ecological and Economic Goals - Marine Parks in the Caribbean. Ambio. 1993;22(2-3):117-25.

102. Sanchirico JN. Marine Protected Areas as Fishery Policy: A Discussion of Potential Costs and Benefits. Washington DC: 2000.

103. Davis D, Tisdell C. Recreational Scuba-Diving and Carrying-Capacity in Marine Protected Areas. Ocean & Coastal Management. 1995;26(1):19-40.

104. Scanlon L, Kull C. Untangling the links between wildlife benefits and community-based conservation at Torra Conservancy, Namibia. Development Southern Africa. 2009;26(1):75-93.

105. Cinner J, McClanahan TR, MacNeil MA, Graham NAJ, Daw TM, Mukminin A, et al. Co-management of coral reef social-ecological systems. Proceedings of the National Academy of Sciences of the United States. 2012;109:5219-22.

106. Arin T, Kramer RA. Divers' willingness to pay to visit marine sanctuaries: an exploratory study. Ocean & Coastal Management. 2002;45(2-3):171-83.

107. Leisher C, Van Beukering P, Scherl LM. Nature's Investment Bank: How Marine Protected Areas Contribute to Poverty Reduction. Arlington, USA: The Nature Conservancy, 2007.

108. Milazzo M, Chemello R, Badalamenti F, Camarda R, Riggio S. The impact of human recreational activities in marine protected areas: what lessons should be learnt in the Mediterranean Sea? Marine Ecology. 2002;23:280-90.

109. Thurstan RH, Hawkins JP, Neves L, Roberts CM. Are marine reserves and non-consumptive activities compatible? A global analysis of marine reserve regulations. Marine Policy. 2012;36(5):1096-104.

110. Oberholzer S, Saayman M, Saayman A, Slabbert E. The socio-economic impact of Africa’s oldest marine park. KODOE. 2010;52:1-9.

111. Bennett NJ, Dearden P. Why local people do not support conservation: Community perceptions of marine protected area livelihood impacts, governance and management in Thailand. Marine Policy. 2014;44:107-16.

112. Roman G, Dearden P, Rollins R. Application of zoning and “Limits of Acceptable Change” to manage snorkeling tourism. Environmental Management. 2007;39:819-30.

113. Adams WM, Aveling R, Brockington D, Dickson B, Elliott J, Hutton J, et al. Biodiversity conservation and the eradication of poverty. Science. 2004;306(5699):1146-9.

114. Francis J, Nilsson A, Waruinge D. Marine protected areas in the eastern African region: How successful are they? Ambio. 2002;31(7-8):503-11.

115. Baglos MC. Integrated coastal management and marine protected areas in the Philippines: Concurrent developments. Ocean and Coastal Management. 2005;48:972-95.

116. Agardy T, Di Sciara GN, Christie P. Mind the gap: Addressing the shortcomings of marine protected areas through large scale marine spatial planning. Marine Policy. 2011;35(2):226-32.

117. Cicin-Sain B, Belfiore S. Linking marine protected areas to integrated coastal and ocean management: A review of theory and practice. Ocean & Coastal Management. 2005;48(11-12):847-68.

118. Salm RV, Clark J, Siirla E. Marine and Coastal Protected Areas: A Guide for Planners and Managers. Washington, USA.: IUCN, 2000.

119. Russ GR, Alcala AC, Maypa AP, Calumpong HP, White AT. Marine reserve benefits local fisheries. Ecological Applications. 2004;14(2):597-606.

120. Gell FR, Roberts CM. Benefits beyond boundaries: the fishery effects of marine reserves. Trends in Ecology & Evolution. 2003;18:448-55.

121. Balmford A, Gravestock P, Hockley N, McClean CJ, Roberts CM. The worldwide costs of marine protected areas. Proceedings of the National Academy of Sciences of the United States of America. 2004;101(26):9694-7.

122. McCrea-Strub A, Zeller D, Sumaila UR, Nelson J, Balmford A, Pauly D. Understanding the cost of establishing marine protected areas. Marine Policy. 2011;35:1-9.

123. Burke L, Bryant D, McManus JW, Spalding M. Reefs at Risk: a map-based indicator of potential threats to the world's coral reefs. Washington, DC: 1998.

124. Bhagwat S, Brown N, Evans T, Jennings S, Savill P. Parks and Factors in Their Success; Letter of response. Science. 2001;293(5532):1045-7.
